# Aurora B kinase is a potent and selective target in MYCN-driven neuroblastoma

**DOI:** 10.18632/oncotarget.6208

**Published:** 2015-10-21

**Authors:** Dominik Bogen, Jun S. Wei, David O. Azorsa, Pinar Ormanoglu, Eugen Buehler, Rajarshi Guha, Jonathan M. Keller, Lesley A. Mathews Griner, Marc Ferrer, Young K. Song, Hongling Liao, Arnulfo Mendoza, Berkley E. Gryder, Sivasish Sindri, Jianbin He, Xinyu Wen, Shile Zhang, John F. Shern, Marielle E. Yohe, Sabine Taschner-Mandl, Jason M. Shohet, Craig J. Thomas, Scott E. Martin, Peter F. Ambros, Javed Khan

**Affiliations:** ^1^ Oncogenomics Section, Genetics Branch, National Cancer Institute, National Institutes of Health, Bethesda, Maryland, USA; ^2^ Children's Cancer Research Institute, St. Anna Kinderkrebsforschung, Vienna, Austria; ^3^ Division of Preclinical Innovation, National Center for Advancing Translational Sciences, National Institutes of Health, Rockville, MD, USA; ^4^ Clinical Translational Research Division, Translational Genomics Research Institute (TGen), Scottsdale, AZ, USA; ^5^ Tumor and Metastasis Biology Section, Pediatric Oncology Branch, National Cancer Institute, National Institutes of Health, Bethesda, MD, USA; ^6^ Texas Children's Cancer Center and Center for Cell and Gene Therapy, Department of Pediatrics, Baylor College of Medicine, Houston, Texas, USA

**Keywords:** neuroblastoma, MYCN, AURKB, high-throughput screening, barasertib

## Abstract

Despite advances in multimodal treatment, neuroblastoma (NB) is often fatal for children with high-risk disease and many survivors need to cope with long-term side effects from high-dose chemotherapy and radiation. To identify new therapeutic targets, we performed an siRNA screen of the druggable genome combined with a small molecule screen of 465 compounds targeting 39 different mechanisms of actions in four NB cell lines. We identified 58 genes as targets, including *AURKB,* in at least one cell line. In the drug screen, aurora kinase inhibitors (nine molecules) and in particular the AURKB-selective compound, barasertib, were the most discriminatory with regard to sensitivity for *MYCN*-amplified cell lines. In an expanded panel of ten NB cell lines, those with *MYCN*-amplification and wild-type *TP53* were the most sensitive to low nanomolar concentrations of barasertib. Inhibition of the AURKB kinase activity resulted in decreased phosphorylation of the known target, histone H3, and upregulation of TP53 in *MYCN*-amplified, *TP53* wild-type cells. However, both wild-type and *TP53* mutant *MYCN*-amplified cell lines arrested in G2/M phase upon AURKB inhibition. Additionally, barasertib induced endoreduplication and apoptosis. Treatment of *MYCN*-amplified/*TP53* wild-type neuroblastoma xenografts resulted in profound growth inhibition and tumor regression. Therefore, aurora B kinase inhibition is highly effective in aggressive neuroblastoma and warrants further investigation in clinical trials.

## INTRODUCTION

Neuroblastoma (NB) is a solid tumor of the peripheral nervous system occurring mostly in infants and younger children. Accounting for 8-10% of all childhood tumors, NB is responsible for 10-12% of cancer-related deaths in children [1, 2]. Although advances in biology-based multimodal treatment strategies have led to an improved outcome for NB patients in the recent decades [2], survival rates for advanced-stage neuroblastoma remain poor ( < 50%) due to the clinical and biological heterogeneity of the cancer and a lack of adequate treatment options [3]. Patients who undergo therapy successfully often suffer from severe, long-term side effects of the aggressive treatment including deafness, cardiac failure, and secondary malignancies [4].

Development of targeted treatment approaches has been challenging due to the heterogeneous nature of this cancer and an insufficient understanding of the biology of high-risk neuroblastoma. The anaplastic lymphoma receptor tyrosine kinase, ALK, is mutated in approximately 10% of all spontaneous cases and currently the only therapeutically-targetable receptor tyrosine kinase in neuroblastoma [5].

In aggressive neuroblastoma, tumorigenesis is largely driven by amplification of the oncogene *MYCN* and/or structural aberrations of the genome, which are difficult to inhibit directly. Recent research has thus been focused on finding surrogate targets that assist MYCN in driving neuroblastoma. Inhibition of either the PI3K signaling pathway or the serine/threonine kinase AURKA was shown to decrease the stability of the MYCN protein and reduce the proliferation of neuroblastoma cell lines and xenografts [6-8]. Both of these targets were consequently evaluated by the pediatric preclinical testing program (PPTP) [9, 10] and the AURKA inhibitor, MLN8237, was advanced to the clinic in single-agent and combination therapy trials (NCT01601535).

In this study, we identified actionable targets in neuroblastoma using a combination of high-throughput RNAi and small molecule drug screens. We found knockdown of the mitotic kinase AURKB and its pharmacological inhibition with barasertib (AZD1152-HQPA) to be highly effective in suppressing neuroblastoma cell growth. Most importantly, *MYCN*-amplified (MNA) cell lines with wild-type TP53 (*MYCN^amp^*/*TP53^wt^*) were highly sensitive to barasertib. Additionally, barasertib effectively decreased tumor volumes and prolonged survival in an MNA mouse xenograft model of human neuroblastoma. Therefore, our studies have uncovered a novel targetable susceptibility in high-risk neuroblastoma with potential clinical application.

## RESULTS

### RNAi screening for actionable vulnerabilities in neuroblastoma cell lines

**Figure 1:**
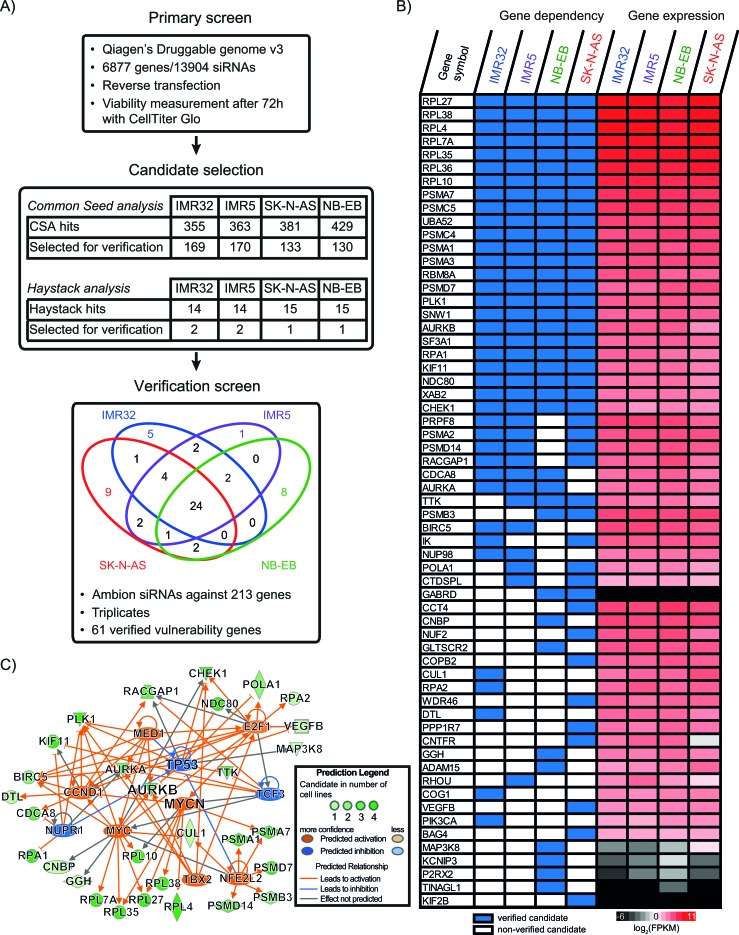
RNAi screening of four neuroblastoma cell lines revealed 61 vulnerability genes **A.** Schematic of the combined RNAi screens and results of the two applied data analysis strategies for candidate gene selection. Venn diagram shows the distribution of verified vulnerability genes. *For complete data see Supplementary table S1.* **B.** Cell dependency for verified vulnerability genes and their expression in the four cell lines used in the screen. The first four columns depict the verification status from the siRNA screens; verified genes are blue, non-verified genes are white. The last four columns are the expression values of these genes in the four cell lines as assessed by RNA sequencing. The color key represents gene expression levels in log2- transformed FPKM values as calculated by RSEM; black coloring indicates low gene expression whereas red coloring represents a high expression. **C.** Predicted relationships between significant upstream expression regulators and vulnerability genes. Negative regulators are colored blue, positive ones are orange. Regulated genes are colored on a green scale based on verification occurrence.

In order to identify genetic vulnerabilities in neuroblastoma, we first performed a high-throughput synthetic lethality screen using a druggable genome siRNA panel that contained 13904 siRNAs to target 6877 genes (Figure 1A, Supplementary table S1). We selected four cell lines from two major genetic subgroups based on the *MYCN*-amplification (MNA) status: two MNA (IMR32 and IMR5) and two non-amplified (SK-N-AS and NB-EB) cell lines. Due to the known off-target binding capabilities of siRNAs, we combined Common Seed Analysis (CSA) [11] with Redundant siRNA Activity (RSA) analysis [12] to minimize false positives during candidate gene selection. In addition, we performed Haystack analysis to detect genes targeted by predicted seed off-target binding [13]. For a broad application in neuroblastoma, we focused on vulnerability genes found in either both cell lines of a genetic subgroup or hits in three or more cell lines. We identified 211 candidate genes by CSA and RSA (130 to 170 genes per cell line), and two additional genes based on Haystack Analysis (Supplementary figure S2A and B). In a confirmatory screen, we verified 61 of 213 genes showing a significant dependency in at least one cell line including 24 genes common in all four (Figure 1A, Supplementary figure S2C). To further reduce potential false-positives, we used RNA sequencing data to ensure candidate gene expression (Figure 1B). Whereas the majority of verified genes were substantially expressed, 3 genes (*GABRD*, *TINAGL1*, and *KIF2B*) were excluded from further consideration due to their low expression. Thus, we identified 58 vulnerability genes whose knockdown resulted in significant reduction of neuroblastoma cell survival.

### Vulnerability genes play a role in cell cycle progression and larger protein complexes

To understand the function of these 58 candidate genes and to discover underlying gene networks including upstream expression regulators, we performed a direct-relationship analysis using IPA (Ingenuity® Systems, www.ingenuity.com). MYCN/MYC and the interacting transcription factor E2F1 were among the strongest expression activators of the vulnerability genes whereas the tumor suppressor TP53 was predicted to be negatively regulated within this network (Figure 1C). Top molecular and cellular functions for these 58 genes were associated with cell death and survival as well as cell cycle regulation (Supplementary figure S3A). More specifically, we identified a dependency on the mitotic kinase AURKB in all four cell lines (Figure 1B and Supplementary figure S4). Target-specific knockdown was confirmed in IMR5(Supplementary figure S4B). As the enzymatic component, *AURKB* partners with two other vulnerability genes found in our screen, *BIRC5*/survivin and *CDCA8*/borealin, in the chromosomal passenger complex (CPC) to drive mitosis (reviewed in [14]). In addition, we discovered regulators and downstream targets of AURKB and the CPC, including CHEK1, PLK1, TTK/Mps1, RACGAP1 and two components of the KNL1/Mis12 complex/Ndc80 complex (KMN), NDC80/HEC1 and NUF2 (reviewed in [14]). Furthermore, we identified additional cell cycle regulators, AURKA and KIF11, as well as numerous subunits of the ribosomal and proteasomal protein complexes (Figure 1B and Supplementary figure S3B). We thus concluded that *AURKB* and the 57 other vulnerability genes were crucial for cell growth and particularly cell cycle progression. In addition, these genes were predicted to be under the transcriptional control of *MYCN/MYC* regulatory network making them potential therapeutic targets in high-risk neuroblastoma.

### Drug screening identified aurora kinase inhibitors as selective compounds for MNA neuroblastoma cell lines

**Figure 2:**
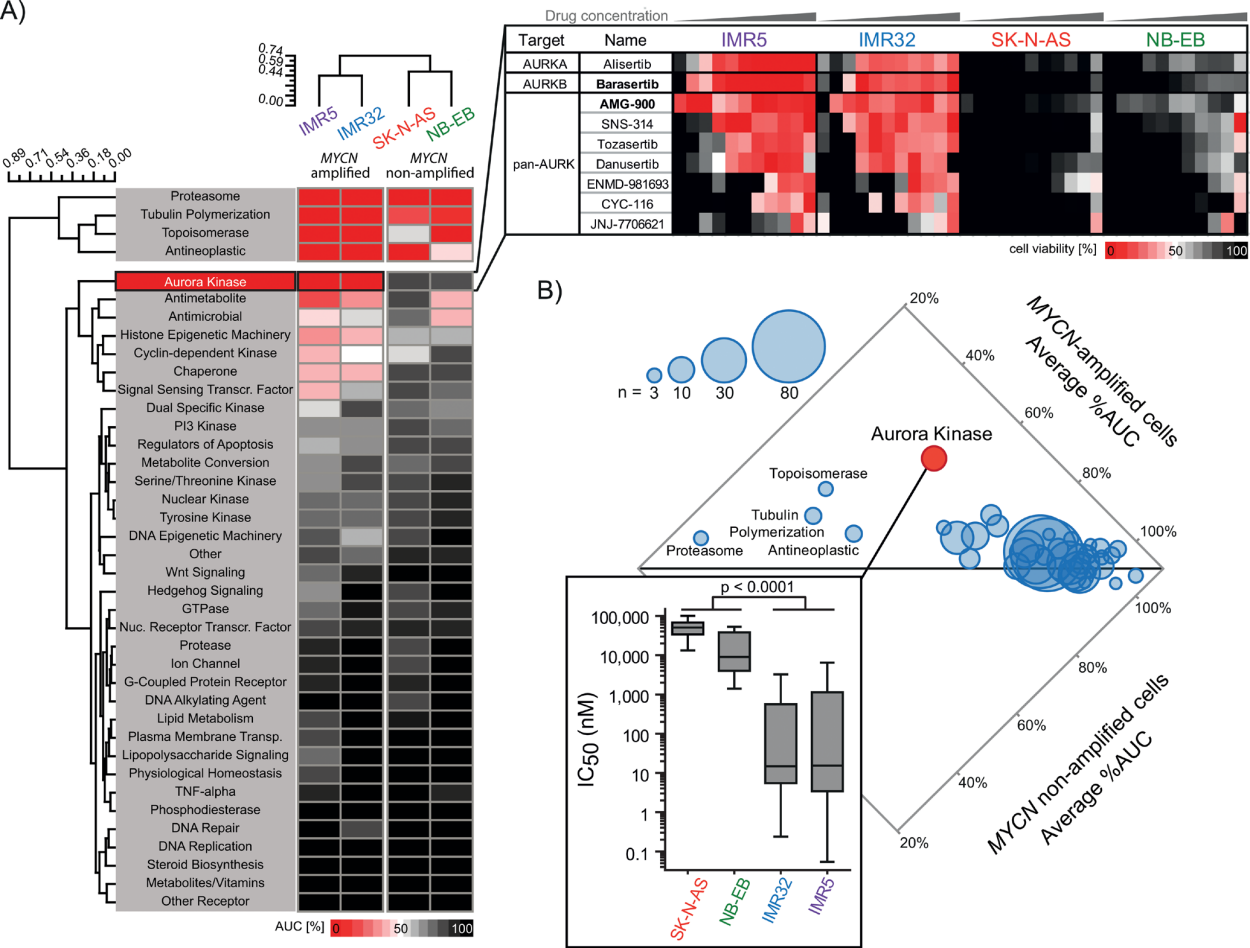
Drug screening of 465 oncology-relevant small molecules demonstrated selective activity of aurora kinases-targeting inhibitors in *MYCN*-amplified neuroblastoma cell lines **A.** Heatmap showing average activities represented by normalized area under the curve (AUC) of the drug response to compound classes categorized by main targeting mechanisms. The dendrograms demonstrate the similarity in drug responses between the four tested cell lines. The expanded inset on the right shows a heatmap of cell viabilities at 48 hours for the ten aurora kinases inhibitors included in the MIPE 3.0 library. **B.** Dot plot comparing the average normalized AUCs of the different compound classes between *MYCN*-amplified cell lines, IMR5 and IMR32, and non-amplified SK-N-AS and NB-EB. Dot sizes represent the number of compounds per class.

In parallel to the siRNA screen, we treated the same four cell lines with 465 small molecules included in the Mechanism Interrogation PlatE (MIPE) compound library [15] to discover novel compounds against neuroblastoma. This collection allowed us to investigate the activity of compounds known to target 39 different processes with relevance in oncology (Supplementary table S2). Consistent with our siRNA screening data, proteasomal inhibitors (3 molecules) were the most active in both subtypes of NB cell lines (Figure 2A). Likewise, inhibitors of other vulnerability genes including *BIRC5*/survivin, *CHEK1*, *KIF11* and *PLK1* demonstrated non-selective activity in either subtype (Supplementary figure S5A), which we confirmed in additional verification tests of a single agent per gene (Supplementary figure S5B, Supplementary table S3). On the contrary, aurora kinase inhibitors (9 molecules) were not only highly active but also most discriminatory regarding *MYCN* amplification (Figure 2A and 2B). This group of aurora kinase inhibitors included molecules preferentially targeting AURKA (i.e. alisertib) or AURKB (i.e. barasertib) as well as both kinases (pan-aurora kinase inhibitors, e.g. AMG-900) (Figure 2A). MNA cell lines responded with an average of 51% area under the curve (AUC with a median IC_50_ of 14.8 nM), compared to an average of 89% AUC in *MYCN-*non-amplified lines (median IC_50_ of 38.3 μM). Therefore, our drug screen demonstrated that vulnerability to aurora kinase inhibition closely linked to with *MYCN* amplification.

### AURKB is a direct transcriptional target of MYCN and high AURKB expression associates with poor outcome in patients with MNA neuroblastoma

**Figure 3:**
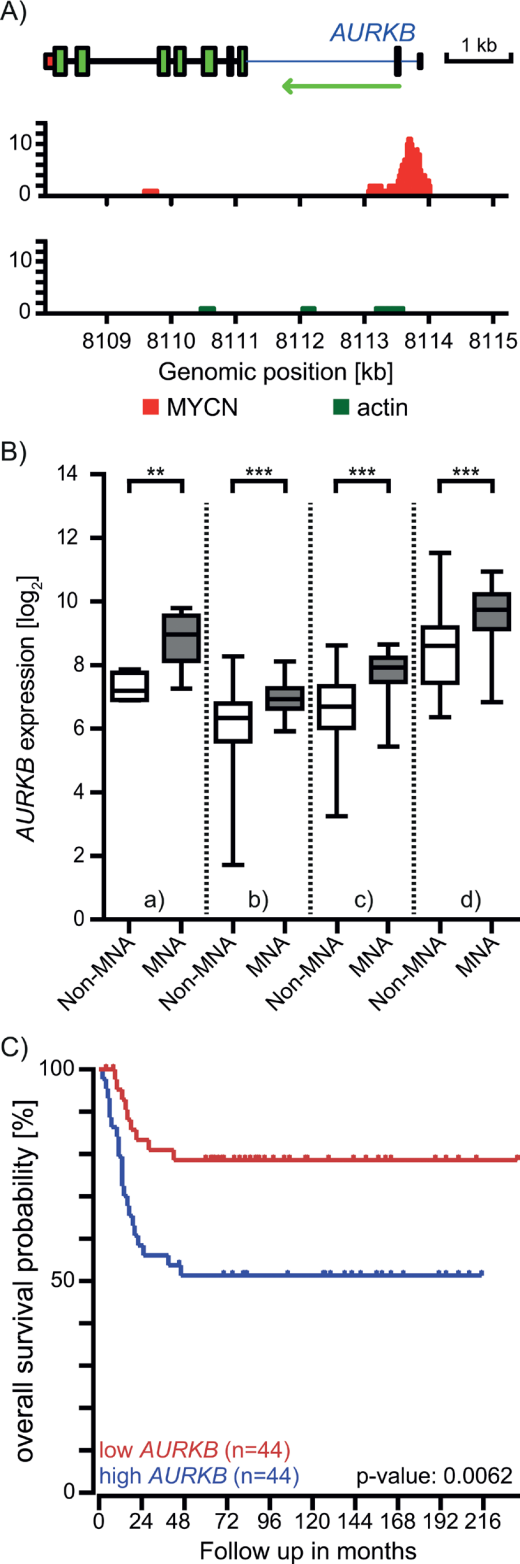
MYCN regulates the expression of the AURKB gene **A.** ChIP sequencing using a MYCN antibody. The MYCN track shows MYCN binding to E-boxes in the promoter region of *AURKB* in MYCN-inducible MYCN3 cells. The negative control antibody against actin showed no enrichment. **B.** Neuroblastomas with *MYCN* amplification (grey) show consistently higher *AURKB* expression in comparison to those without MNA (white) based on the NB cell line panel (Pediatric Tumor Affymetrix Database at https://pob.abcc.ncifcrf.gov/cgi-bin/JK, (a, *n* = 10)) and publically available microarray data for three independent patient cohorts (GSE16476 (b, *n* = 88), GSE3960 (c, *n* = 101), GSE19274 (d, *n* = 100), http://r2.amc.nl). Box-and-whisker blots are based on the most discriminatory *AURKB* probe. **C.** Kaplan-Meier curves based on the expression of *AURKB* in NB patient tumors (GSE16476, *n* = 88, http://r2.amc.nl).

Selective sensitivity based on *MYCN* status was recently shown for the AURKA inhibitor MLN8237 in neuroblastoma [16] which is due to the stabilizing effect of AURKA on MYCN [7, 8]. However, neither enhanced expression nor depletion of AURKB showed a comparable effect on MYCN protein level [16]. Conversely, MYCN expression induction correlated with AURKB expression [17]. To investigate if MYCN directly regulates AURKB expression, we first examined if MYCN binds to the *AURKB* promoter by chromatin immuno-precipitation sequencing (ChIP-seq) in an NB cell line with an inducible MYCN-expression construct [18]. We detected a prominent MYCN binding at the promoter region of the *AURKB* gene indicating that MYCN regulates AURKB expression directly (Figure 3A). In accordance, MNA NB cell lines and tumors consistently express significantly higher levels of AURKB than non-amplified counterparts (Figure 3B). These evidences suggested that AURKB is a direct transcriptional target of MYCN. Furthermore, patients with a high AURKB expression have a significantly worse prognosis for overall survival (Figure 3C), suggesting that AURKB is a potential target for patients with MNA neuroblastoma.

### *MYCN*-amplified/TP53 wild-type neuroblastoma cell lines are highly sensitive to AURKB inhibition by barasertib

**Figure 4:**
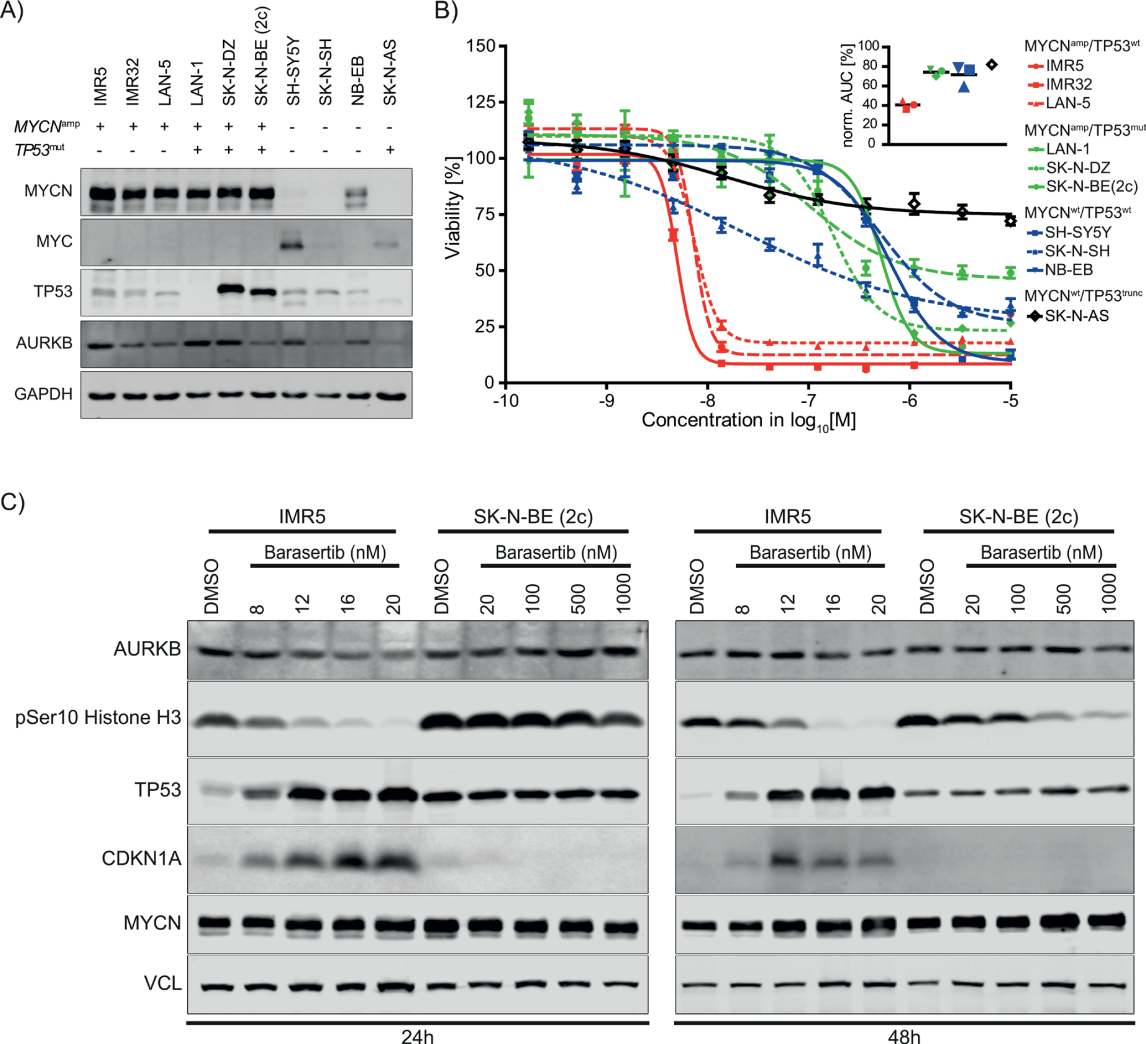
AURKB expression in cell line panel and drug response to barasertib **A.** Western blots showing AURKB, MYCN, MYC, and TP53 in the NB cell line panel. **B.** Dose response curves to barasertib at 72h of incubation normalized to the DMSO control sample (mean ± SD, *n* = 5). The inset depicts the normalized AUCs of each response curve. **C.** Downstream effects of barasertib-induced AURKB inhibition on histone H3 phosphorylation and TP53 protein levels in IMR5 and SK-N-BE (2c) as indicated after 24h (left) and 48h (right). TP53 and its downstream effector CDKN1A were up-regulated by barasertib treatment in a dose responsive manner in IMR5 cells, but not in SK-N-BE (2c) cells.

In our drug screen, barasertib was highly selective and effective in IMR5 and IMR32 cells inhibiting growth in low nanomolar concentrations with an almost complete maximum response rate (Figure 2A). A recent report, which suggested that AURKB inhibition by barasertib stabilizes TP53 in breast cancer [19], led us to hypothesize that the sensitivity to AURKB inhibition in MNA NB cells is mediated by intact TP53 activity. Therefore, we examined the response to barasertib in an expanded NB cell line panel including three TP53-mutant MNA cell lines, LAN-1, SK-N-DZ, and SK-N-BE (2c), and non-MNA, TP53^trunc^ SKNAS (Table 1). Western immunoblotting confirmed general expression of AURKB and MYCN/MYC albeit at different levels (Figure 4A). TP53 was detected in all cell lines except for LAN-1 and SK-N-AS which carry a deleterious *TP53* mutation (546C>A) and a homozygous deletion of exons 10 and 11, respectively. In contrast, *TP53*-mutants SK-N-DZ and SK-N-BE (2c) exhibited the highest protein levels of TP53, which is likely due to an altered stability of the mutant protein. Barasertib elicited a similarly strong response in the *MYCN^amp^*/*TP53^wt^* cell line LAN-5 as in IMR5 and IMR32 (Figure 4B, Table 1). In contrast, *MYCN^amp^*/*TP53^mut^* cell lines and *MYCN*-non-amplified cell lines (NB-EB, SK-N-SH and SH-SY5Y) were substantially less sensitive. Interestingly, *MYCN*-non-amplified SK-N-AS which lacks TP53 expression was highly resistant to the maximum dose of barasertib and thus featured the highest normalized AUC of all cell lines. These data indicated that AURKB inhibition by barasertib was far more effective in MNA NB cells with wild-type TP53.

**Table 1:** Neuroblastoma cell lines selected for testing of barasertib in drug response assay

Cell line	*MYCN* status	TP53 status	Viability IC_50_ [nM]	Norm. AUC of viability curve [%]	Caspase 3/7 activity EC_50_ [nM]
IMR32	amplified	wild-type	4.99	36.40	2.4
IMR5	amplified	wild-type	7.28	40.75	5.51
LAN-5	amplified	wild-type	7.35	44.93	5.08
LAN-1	amplified	546C>A (C182X)	522.4	76.94	628.06
SK-N-BE (2c)	amplified	404G>T (C135F)	104.47	75.33	86.9
SK-N-DZ	amplified	329G>T (R110L)	171.79	70.21	134.28
NB-EB	non-amplified	wild-type	523.6	79.30	251.19
SH-SY5Y	non-amplified	wild-type	633.87	76.59	320.63
SK-N-SH	non-amplified	wild-type	22.39	58.75	495.45
SK-N-AS	non-amplified	Hom. del. of exons 10 and 11	15.45	82.02	13.87

Area under the viability curve was normalized against the area under the upper asymptote of the fitted curve as estimated by GraphPad Prism 5.

### Barasertib upregulates TP53 and CDKN1A in MNA NB cells with wild-type TP53

In order to examine the downstream effect of AURKB inhibition in NB cells, we performed immunoblotting on protein lysates of cells treated with different concentration of barasertib. We found that cell lines responded to AURKB inhibition with time- and concentration-dependent loss of phosphorylation of histone H3, an AURKB downstream target (Figure 4C). Furthermore, barasertib treatment induced TP53 protein expression and its downstream target CDKN1A in *TP53* wild-type IMR5 cells, whereas *TP53* mutant SK-N-BE (2c) showed neither an induction of TP53 nor CDKN1A. Inhibition of AURKB did not change protein levels of MYCN. Therefore, these data indicate that upregulation of TP53 and its downstream target CDKN1A resulting from inhibition of AURKB likely induces cell death in *MYCN*^amp^/*TP53*^wt^ neuroblastoma cells.

### Barasertib induces G2/M arrest and apoptosis regardless of TP53 status

**Figure 5:**
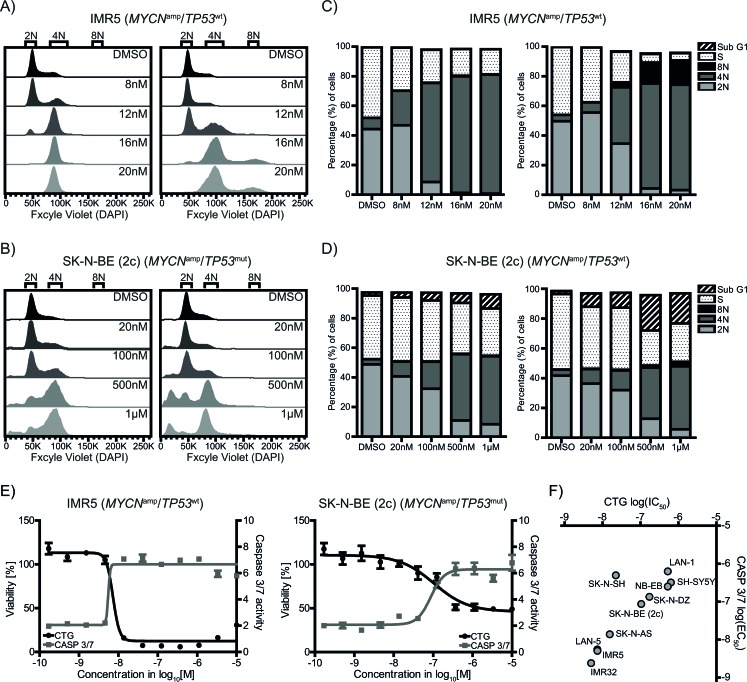
AURKB inhibition by barasertib leads to G2/M arrest and induction of caspase 3/7 in neuroblastoma cell lines Flow cytometric analysis of the dose-dependent effect on cell cycle progression in IMR5 **A.** and SK-N-BE (2c) **B.** Profiles of the cell cycle distribution are representatives of at least three independent experiments. The column charts for IMR5 **C.** and SK-N-BE (2c) **D.** depict the percentages of cells in the different cell cycle phases after 24h (left) and 48h (right) treatment with barasertib. EdU-positivity marks cells actively replicating DNA during S phase with varying DNA content (dotted fill). EdU-negative cells contain integer DNA content and are labeled according to ploidy, 2N (light grey), 4N (dark grey), and 8N (black). Colum segments for cells in Sub G1 are striated. **E.** Inverse association between decreased viability (black curve) and caspase 3/7 activity (grey curve) for IMR5 (left) and SK-N-BE (2c) (right) after 72h of treatment with barasertib. **F.** log_10_(IC_50_) of viability (CTG) plotted against log_10_(EC_50_) of caspases 3/7 (CASP 3/7) activation after 72h.

As AURKB plays a crucial role during M-phase progression and chromosome segregation, we investigated the effect of barasertib on the cell cycle and DNA content in one of the most sensitive cell lines, IMR5 and the less-sensitive *MYCN*^amp^/*TP53*^mut^ SK-N-BE (2c). Barasertib induced a profound G2/M-arrest as well as polyploidization, albeit at different concentrations, in both cell lines as determined by flow cytometry (Figure 5A-5D). In accordance with the drug response assays, 20nM of barasertib resulted in significant cell cycle arrest in IMR5 cells, but exhibited only a minor effect in SK-N-BE (2c). Polyploidization was only detectable in a small percentage of SK-N-BE (2c) cells likely due to a slower proliferation rate compared to IMR5. It is well known that persistent G2/M-arrest as well as polyploidization can lead to induction of apoptosis [20, 21]. In both IMR5 and SK-N-BE (2c), increasing concentrations of barasertib induced caspases 3 and 7 (Figure 5E). The inverse relationship of decreased viability and increased caspase 3/7 activation was observed in all neuroblastoma cell lines with a linear correlation between the half maximal inhibitory (IC_50_) and effective (EC_50_) concentrations of the response curves, respectively (Figure 5F). Thus, barasertib can induce cell cycle arrest and apoptosis in *MYCN*^amp^/*TP53*^wt^ cells at much lower drug concentrations than in *TP53*-mutant and *MYCN*-non-amplified neuroblastoma cells.

### Barasertib suppresses tumor growth in a neuroblastoma mouse xenograft model

**Figure 6:**
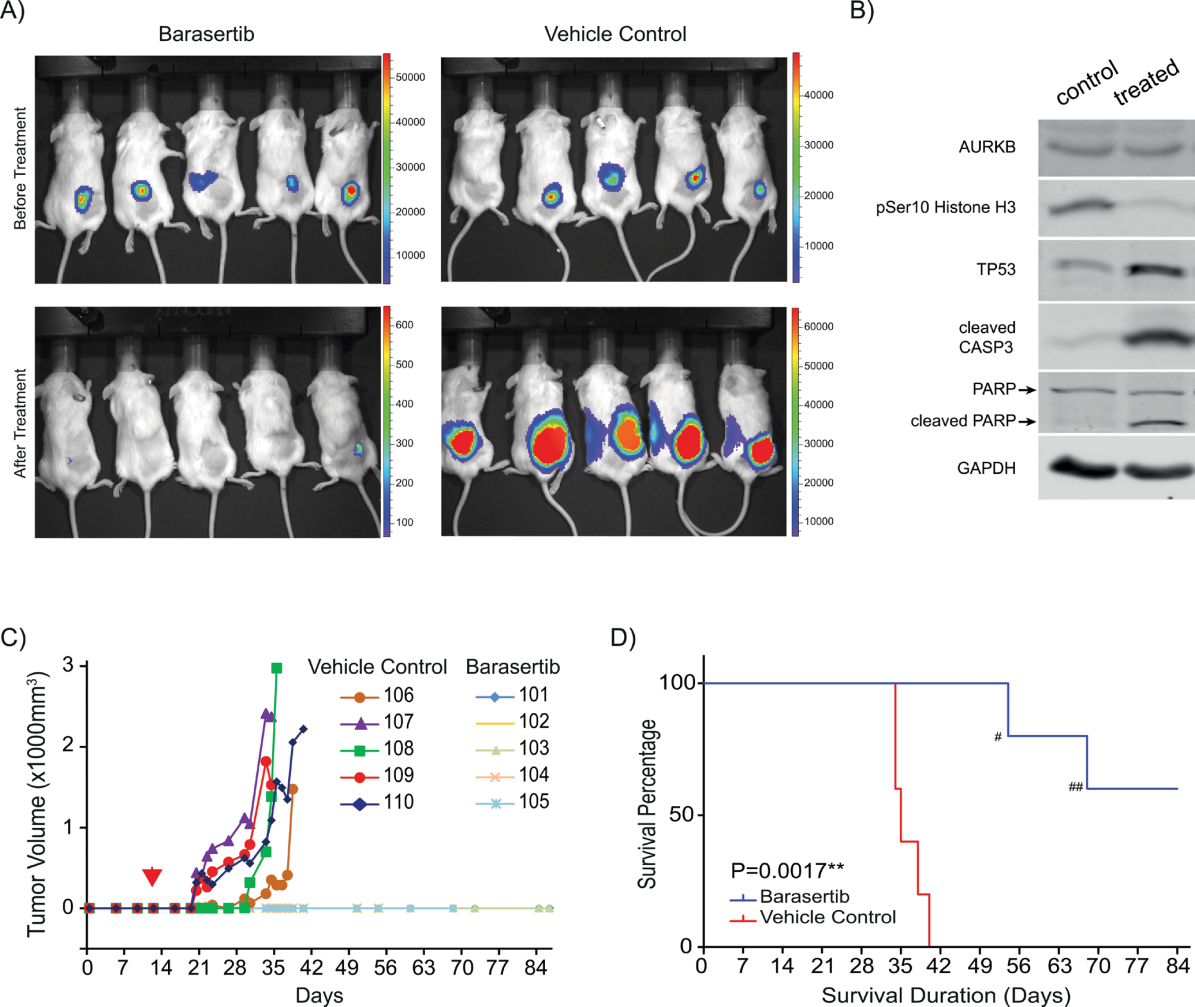
Barasertib inhibits tumor formation in an IMR32 xenograft model **A.** Imaging of vehicle-treated control and barasertib-treated NSG mice before the treatment (upper panel) and after treatment (lower panel). **B.** Immunoblotting demonstrated that AURKB inhibition by barasertib resulted in reduction of phosphorylation of its downstream target histone H3 and increased TP53 expression as well as induction of apoptosis in representative IMR32 xenografts. **C.** *In vivo* tumor growth. Tumor volume was calculated based on size measurements by caliper. Red arrowhead marks the start of treatment. **D.** Survival curve of mice. Survival is defined as reaching the endpoint of the experiment (2cm-diameter of tumor). ^#^and ^##^ mark tumor-unrelated deaths.

In order to examine the *in vivo* efficacy of barasertib to treat MNA neuroblastoma, we used *MYCN^amp^*/*TP53^wt^* IMR32 cells expressing luciferase in a xenograft pre-clinical mouse model. Ten mice with grafted tumors were randomized into two groups: one treated with barasertib and the other with vehicle control starting 12 days after tumor grafting (Figure 6A). Barasertib significantly suppressed tumor growth *in vivo* (Figure 6A and 6C). Target-specific inhibition of AURKB led to a decreased phosphorylation of histone H3 at Ser10 and an induction of TP53 as well as activation of apoptosis as indicated by the cleavage of caspase-3 and PARP (Figure 6B). Due to the suppression of tumor growth, mice in the barasertib-treated group demonstrated a significantly increased probability for survival (*P* = 0.0017) (Figure 6D). Despite using reported dosing [22, 23], two mice died of potential drug side effects (possible anemia and intestinal ileus) without any detectable tumors. The *in vivo* study thus demonstrated the potential efficacy of barasertib to treat NB with *MYCN* amplification.

## DISCUSSION

In this study, we identified *AURKB* and 57 other essential genes for neuroblastoma cell survival by interrogating an siRNA library focused on target druggability. The importance of AURKB was demonstrated by the decrease of cell viability after *AURKB* knockdown in 4 NB cell lines and supported by our discovery of numerous vulnerability genes which interact with the mitotic kinase and the CPC. By screening a collection of oncology-relevant small molecules in parallel, we identified the AURKB-selective inhibitor barasertib and seven other classes of agents that specifically inhibit other vulnerability genes discovered in the RNAi experiments. Whereas AURKA, BIRC5, CHEK1, KIF11, PLK1 and the proteasome are currently evaluated in clinical trials or were preclinically examined by the PPTP and other groups [10, 24-28], AURKB inhibition was only suggested to be a potential target for NB tumor initiating cells [29] but has not been advanced to *in vivo* testing in neuroblastoma.

In this study, we demonstrate that barasertib is efficacious in a panel of neuroblastoma cell lines with a preference for the *MYCN*-amplified and TP53 wild-type subtype. Previously, synthetic lethality between barasertib and members of the Myc family of transcription factors was shown in *MYC*-amplified small cell lung cancer cell lines and MYC-overexpressing RPE cells [30, 31]. In addition, pan-aurora kinase inhibitors, VX-680 and CCT137690 which also target AURKB, were reported to sensitize cancer cells with high expression of MYC and MYCN [31, 32]. Nevertheless, the underlying relationship between MYCN and AURKB has not been investigated in detail. Consistent with the report that induction of high level MYCN leads to the upregulation of AURKB expression [17], our ChIP-Seq data demonstrated that MYCN directly controls AURKB expression by binding to E-box motives in the promoter region. In contrast to AURKA which regulates MYCN protein stability directly [7], neither *AURKB* knockdown nor overexpression were shown to affect MYCN protein levels [16] which is consistent with our data on AURKB inhibition.

MYCN induces the expression of the tumor suppressor TP53 [33] which is likely a reason for the high rate of apoptosis reflected by the high mitosis-karyorrhexis index of MNA neuroblastoma [34]. Whereas more than 50% of adult tumors possess mutations in the TP53 pathway, it is only mutated in 2% of neuroblastoma at diagnosis but the mutation rate rises in relapse tumors after chemotherapy [35, 36]. Counterbalancing the general activeness of TP53, MYCN was also shown to induce the expression of the E3 ubiquitin-ligase and TP53-suppressor, MDM2 [37], which is often amplified in neuroblastoma [38]. A recent report demonstrated that AURKB can directly phosphorylate TP53, thus priming the suppressor for ubiquitination upstream of MDM2 [19]. Accordingly, our data demonstrated upregulation and activation of TP53 as a result of AURKB inhibition which suggests that AURKB contributes to MYCN-mediated repression of TP53 function in neuroblastoma.

The effect of TP53 on AURKB function is less well-understood but it was suggested that activity of AURKB inhibitors depends on the *TP53* mutational status [20, 39]. TP53-deficiency was reported to promote mitotic slippage in barasertib-treated cells [39] as well as apoptosis and endoreduplication upon treatment with the pan-aurora kinase inhibitor VX-680 [20]. Others found no synthetic lethality between mutant TP53 and aurora kinase inhibition [31]. In our study, *TP53* status showed no correlation with sensitivity to barasertib by itself but in combination with *MYCN* amplification, *TP53*^wt^ cell lines were significantly more sensitive.

We found that barasertib induced upregulation of TP53 and CDKN1A protein levels in a dose-dependent manner in *TP53*^wt^ IMR5 which, despite CDKN1A induction, arrested in G2/M phase contrary to an expected G1/S phase arrest. However, MYCN-mediated overwriting of the TP53 post-mitotic checkpoint has been commonly observed in *MYCN*-amplified/wt-*TP53* neuroblastoma cell lines [40, 41]. Mutant TP53 was unaffected in SK-N-BE (2c) suggesting that AURKB has a different intrinsic effect on the mutant protein. Nevertheless, both cell lines underwent endoreduplication resulting in polyploidy and exhibited signs of apoptosis signaled by induction of caspases 3 and 7.

In a variety of solid tumors and leukemias, barasertib has shown significant tumor growth inhibition when used preclinically as monotherapy and when combined with standard chemotherapeutic agents or ionizing radiation [22, 42-49]. Phase I clinical trials in patients with acute myeloid leukemia and solid tumors attested good tolerability with manageable toxicities [50-54]. However, reports on the efficacy of barasertib are conflicting in the two completed phase 2 studies in different types of cancer [55, 56]. In this study, barasertib caused profound inhibition of tumor growth in *MYCN*-amplified IMR32 xenografts. Despite the potential treatment-related death of two mice, the drug-treated group showed complete tumor suppression and remained tumor-free throughout the trial, which warrants future studies to use this compound for neuroblastoma with wild-type TP53 and *MYCN* amplification.

In conclusion, our study demonstrated the importance of AURKB for neuroblastoma cell survival and the association of high *AURKB* expression with poor prognosis for neuroblastoma patients. We further showed an AURKB inhibitor barasertib is more efficacious in neuroblastoma with *MYCN* amplification and wild-type *TP53*. Finally, we demonstrated barasertib significantly suppressed tumor growth in a human neuroblastoma xenograft mouse model. Therefore, our study established AURKB as a rational target for patients with highly aggressive *MYCN*-amplified, TP53 wild-type neuroblastoma.

## MATERIALS AND METHODS

### Cell lines and cell culture

*MYCN*-amplified (MNA) cell lines, IMR32, IMR5, LAN-1, LAN-5, SK-N-BE (2c), SK-N-DZ, as well as the *MYCN*-non-amplified cell lines SK-N-AS, NB-EB, SK-N-SH and SH-SY5Y were verified by short tandem repeat (STR) analysis. SK-N-DZ and SK-N-BE (2c) were cultured in DMEM+ GLUTAMAX (Life Technologies, NY) and all other cell lines in RPMI medium 1640 (1x) + GLUTAMAX (Life Technologies, NY) medium supplemented with 10% fetal bovine serum or fetal calf serum and the antibiotics penicillin and streptomycin.

### High-throughput siRNA screening and AURKB knockdown

The druggable genome siRNA library targeting 6877 genes with two or more siRNAs/gene was obtained from Qiagen (version 3, Valencia, CA). For the verification screen, siRNAs targeting candidate genes, including *AURKB*, were obtained from Ambion (Carlsbad, CA). Non-silencing negative (Silencer Select Negative Control No. 2) and lethal positive siRNA controls were obtained, respectively, from Ambion and Qiagen.

The high-throughput RNAi discovery screen was performed using reverse transfection as described previously [57]. Briefly, 384-well siRNA library assay plates were preprinted from the manufacturer's plates, frozen and thawed on the day of the assay. RNAiMAX transfection reagent (Invitrogen, Carlsbad, CA) was diluted in OptiMEM (Invitrogen, Carlsbad, CA) at a 6:1 lipid:siRNA (volume:weight) ratio for IMR5 and IMR32 or an 8:1 ratio for SK-N-AS and NB-EB. Neuroblastoma cells were resuspended in growth media supplemented with 20% fetal bovine serum and plated at sub-confluent concentrations after 96h. Plates were incubated at 37˚C and 5% CO_2_ for 96 hours. Dimethyl sulfoxide (DMSO) at a final in-plate concentration of 0.1% was added after 24h.

For verification screening, daughter plates with 0.8 μmol of siRNA in 2 μl DMSO were printed from the master plates obtained from Ambion. Control siRNAs were added manually to each assay plate. RNAimax was diluted in RPMI. Cells were added in RPMI with 20%FBS (2000 cells per well for IMR5, IMR32, and NB-EB; 1500 cells for SK-N-AS). In both screens, CellTiter Glo (Promega, Madison, WI) was used to measure total cell viability in relative luminescence units (RLU) with an EnVision plate reader (Perkin-Elmer, Wellesley, MA).

For both screens, cell viability was measured by CellTiter Glo (Promega, Madison, WI) 96h after siRNA transfection.

For the confirmation of aurora B kinase-specific knockdown, cells were reverse transfected under the same conditions as the verification screen with three aurora B Silencer Select siRNAs (s17611, s17612 and s17613, Ambion, Carlsbad, CA), independently.

### Data analysis for candidate selection

Raw data obtained from the discovery screen were first corrected for plate variability by normalizing against the non-transfected reference wells. Robust Z-scores for each siRNA were calculated [58]. A modification of the Common Seed analysis (CSA) as proposed by Marine et al. was applied to take potential off-target effects into consideration [11]. In short, siRNAs from the Qiagen library were grouped based on matching seed sequences. The activity of each of the members was corrected by the median activity of all siRNAs in a group. After median-seed correction, the collection of siRNA activities were fit to symmetrical super Gaussian distributions with calculated kurtosis around 6 (range 5.9-6.4) and skewness around 1.6 (range 1.5-1.6) for each cell line to estimate significance thresholds (Supplementary figure S1). Assuming that the data followed an exponential distribution (kurtosis = 6 and skewness = 2), significance cutoffs for a p-value = 0.05 were estimated based on a cumulative distribution function. Cutoffs for the lower tail of the distributions were −1.15 (IMR32), −1.27 (NB-EB), −1.29 (IMR5), and −1.06 (SK-N-AS). Redundant siRNA activity (RSA) analysis was applied on the median-seed corrected siRNAs to take the activities of all siRNAs targeting the same gene into consideration [12]. A rank distribution was determined for the adjusted activities of all siRNAs and log_10_p-values assigned to the corresponding genes based on an iterative hypergeometric distribution formula. Genes targeted by siRNAs significantly distributed at the top of the ranking (log_10_p < −1.301) as determined by RSA and with at least one siRNA active below the estimated significance cutoff were selected as potential hits for each cell line.

To employ the knowledge on seed-based off-target binding, Haystack analysis was implemented to identify 3′-UTR regions of genes containing complementary sequences to the most active seeds [13]. Only genes with a significant negative estimate were considered potential candidates.

Analysis of the verification screen data was adapted to the expectation that a high number of siRNAs would be active in each cell line. The 639 siRNAs were normalized by subtracting the positive control and dividing by the negative control. The relative viabilities were then ranked by RSA similar as described for the discovery screen. Genes with a log_10_p-value < −1.301 were considered verified.

### Expression analysis

Cells were plated in 6-well plates and treated with DMSO after overnight incubation. RNA was isolated from harvested cells using the Trizol method and the QIAGEN All Prep DNA/RNA mini kit (Hilden, Germany) according to the manufacturer's protocol. In short, Trizol was added to the plates and the cell lysates transferred into tubes. Chloroform was added for phase separation. The aqueous phase was transferred to a fresh tube and 70% ethanol was used to precipitate the RNA before applying the mixture to Mini RNeasy columns for filtration. RNA was quantified with a Nanodrop spectrophotometer and RNA integrity was measured with an Agilent 2100 Bioanalyzer.

Library preparation for PolyA-selected mRNA sequencing was performed according to Illumina's TruSeq RNA sample preparation v2 guide (Illumina®, San Diego, CA). Libraries were sequenced on an Illumina HiSeq2500 using the 100-bp paired-end sequencing protocol. After fastq files were generated using CASAVA (Illumina®, San Diego, CA), RSEM was applied to estimate the expression levels of each gene using the hg19 reference transcriptome [59]. Expression was calculated in fragments per kilobase of exon per million fragments mapped (FPKM). A smoothing factor of 0.6 was added to the expected count and of 0.01 to FPKM values before log-transformation.

### MYCN-ChIP sequencing

ChIP sequencing experiments were performed using the Tet-inducible MYCN3 cell line using doxycycline (1 mg/ml) for MYCN induction as previously described [18]. Actin served as a negative control. Reads were mapped against the human reference genome (hg19) and stored using the Genboree discovery platform. Visualization of MYCN binding to regulatory DNA sequences, especially E-box motifs was conducted using the UCSC genome browser (hg19).

### High-throughput drug screening assay and nonlinear curve fitting

The high-throughput screen of the Mechanism Interrogation PlatE (MIPE) compound library to identify small compound inhibitors was conducted according to Mathews *et.al.* [15, 60]. A full list of the compounds and vendors is available from the authors upon request. The main mechanism of action for each compound was annotated based on suppliers’ information. Briefly, for each cell line, 1000 cells were dispensed in their standard growth media with a Multidrop dispenser into 1536-well plates and allowed to settle overnight. Plates were then treated with DMSO followed by the immediate addition of bortezomib (control compound) or library compounds. For the 11-point dilution series, library compounds were diluted 1-to-3 starting with an in-plate concentration of ∼38.3 μM. After an incubation period of 48h at 37˚C with 5% CO_2_, viability was measured with CellTiter Glo (Promega, Madison, WI) using a ViewLux imager (Perkin Elmer, Wellesley, MA). Relative luminescence units (RLU) for each well were normalized to the median RLUs of the DMSO control wells set as 100% viability and median RLUs of the 2 mM-bortezomib control wells as 0% viability. Normalized dose responses were fitted to a 4-parameter nonlinear model applying a custom gird-based algorithm that can handle outliers [61]. Retesting of selected compounds was conducted in 384-well plates with 1000 cells per well. Drugs were added in an 11-point dilution series (1-to-3) plus DMSO. Viability was assessed after 72h as described for the siRNA verification screen. All drugs for retesting were obtained from Selleck USA (Houston, TX).

### Antibodies and immunoblotting

For immunoblotting, the samples were harvested by trypsinization and lysed in 2x SDS-loading buffer and PBS. DNA was sheared by sonication. The antibodies used were as follows: phospho-Ser10-Histone H3 (Cat.# 9715), AURKB (Cat.# 3049) and Cleaved Caspase-3 (Cat.# 9661) from Cell Signaling, TP53 (Cat.# sc-126) and GAPDH (Cat.# sc-32233) from Santa Cruz Biotechnology, MYCN (Cat.# OP13L) and CDKN1A (p21/WAF1) (Cat.# OP64) from Calbiochem (San Diego, CA), vinculin (Cat.# V9131) from Sigma-Aldrich, and PARP (Cat.# 556494) from BD Biosciences. Secondary antibodies were obtained from LI-COR (goat anti-mouse; Cat.# 926-32210) and Thermo Scientific Pierce (goat anti-rabbit; Cat.# SA5-35571). The Odyssey Imaging System from LI-COR was used for signal detection.

### Flow cytometry

IMR5 and SK-N-BE (2c) were plated into T25 flasks for cell cycle analysis and allowed to attach overnight at subconfluent concentrations after 48h. IMR5 cells were treated with 8 nM, 12 nM, 16 nM and 20 nM of barasertib whereas SK-N-BE (2c) cells were treated with 20 nM, 50 nM, 100 nM, 500 nM and 1 μM of the compound. The Click-iT EdU Alexa Fluor 647 Flow Cytometry Assay Kit (Life Technologies, Eugene, OR) was used for cell cycle assessment according to the manufacturer's protocol. In short, cells were treated with 10 μM EdU (1:1000) for 2h before collection after 24h and 48h. Cells were fixed, permeabilized, and washed before incubating the samples with the Click-iT reaction cocktail for 30min at RT. FxCycle Violet (Life Technologies, Eugene, OR) was used to counterstain DNA. Flow cytometric analysis was performed on a BD LSRFortessa Cell Analyzer (Becton Dickinson, San Jose, CA). Data were acquired with the BD FACSDiva software and analyzed with FlowJo version 10 (FlowJo, LLC, Ashland, OR).

### Xenograft studies

A human neuroblastoma xenograft mouse model was used to examine the efficacy of barasertib for treating *MYCN*-amplified tumors. Animal studies were performed with 4- to 6-week-old female NOD scid gamma (NSG) mice (Taconic, Hudson, NY). Five million IMR32 neuroblastoma cells stably expressing luciferase in 100 μl 1:1 HBSS:Matrigel solution (Cultrex® 3D culture matrix reduced growth factor basement membrane extract PathClear®, Trevigen, Gaithersburg, MD) were subcutaneously engrafted into the right flank of each mouse to generate an *in vivo* neuroblastoma xenograft model. Barasertib was obtained through the Developmental Therapeutics Program, the Division of Cancer Treatment Diagnosis and Centers, National Cancer Institute. Barasertib was dissolved in a solution vehicle made of 30% PEG400, 0.5% Tween 80, 5% Propylene glycol (Sigma-Aldrich, St. Louis, MO), and administrated to the mice via intraperitoneal injection at 50mg/kg four times a week for two consecutive weeks. Mice were monitored weekly by palpation twice and luciferase imaging using a Xenogen VivoVision IVIS 100 system (Caliper Life Science, Hopkinton, MA). Tumor size was measured by caliper twice a week and tumor volume was calculated using this formula: (long axis x short axis^2^)/2. The endpoint of the experiment was any tumor axis reaching 20mm. To investigate target-specific effects of barasertib *in vivo*, mice of the control arm were treated with either a cycle of four daily doses of 50mg/kg barasertib or vehicle control immediately after the endpoint of the tumor growth study was reached. Protein isolation from these tumors for immunoblotting was done in RIPA-buffer containing phosphatase inhibitors (sodium orthovanadate and NaF) and protease inhibitors (Sigma-Aldrich, St. Louis, MO) using a gentleMACS Dissociator (Miltenyi, San Diego, CA) for tissue homogenization.

## SUPPLEMENTARY MATERIAL FIGURES AND TABLES




