# Supplementary material for: Aurora B kinase is a potent and selective target in MYCN-driven neuroblastoma
Source: Oncotarget. 2015 Oct 21;6(34):35247–62. doi: 10.18632/oncotarget.6208 (PMC4742102; doi:10.18632/oncotarget.6208)
Supplement: Supplementary file 1 [file oncotarget-06-35247-s001.pdf]

Aurora B kinase is a potent and selective target in MYCN-driven neuroblastoma

Supplementary Material

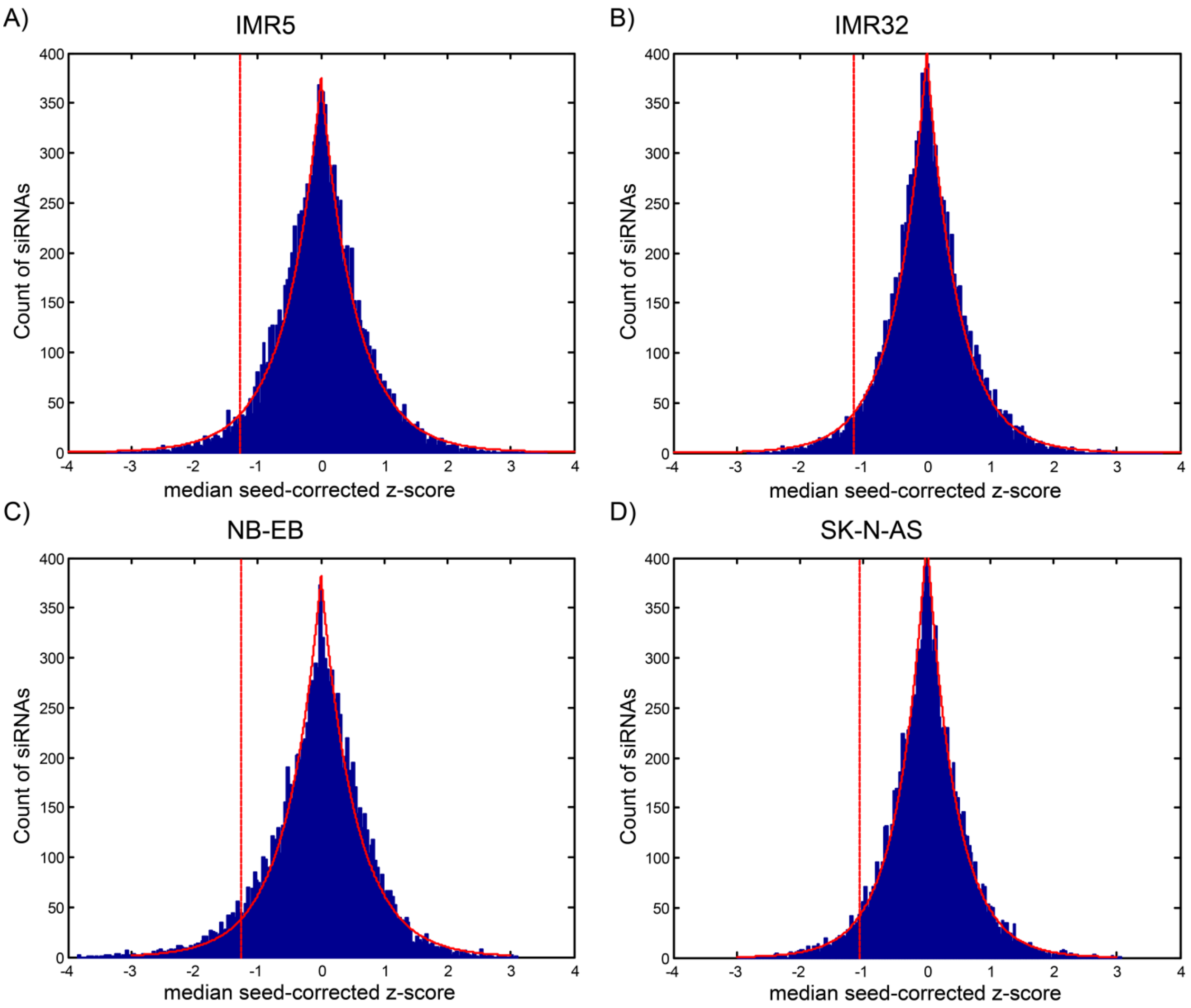

Supplementary figure S1: Estimated significance thresholds for siRNAs in druggable genome screen. Density distribution of seed-corrected siRNAs in the discovery screen for the respective cell line (dark blue), IMR5 **(A)**, IMR32 **(B)**, NB-EB **(C)** and SK-N-AS **(D)**. The red solid line depicts the exponential curves that were fit to

the data; the red dashed line represents the calculated significance cutoff for each dataset.

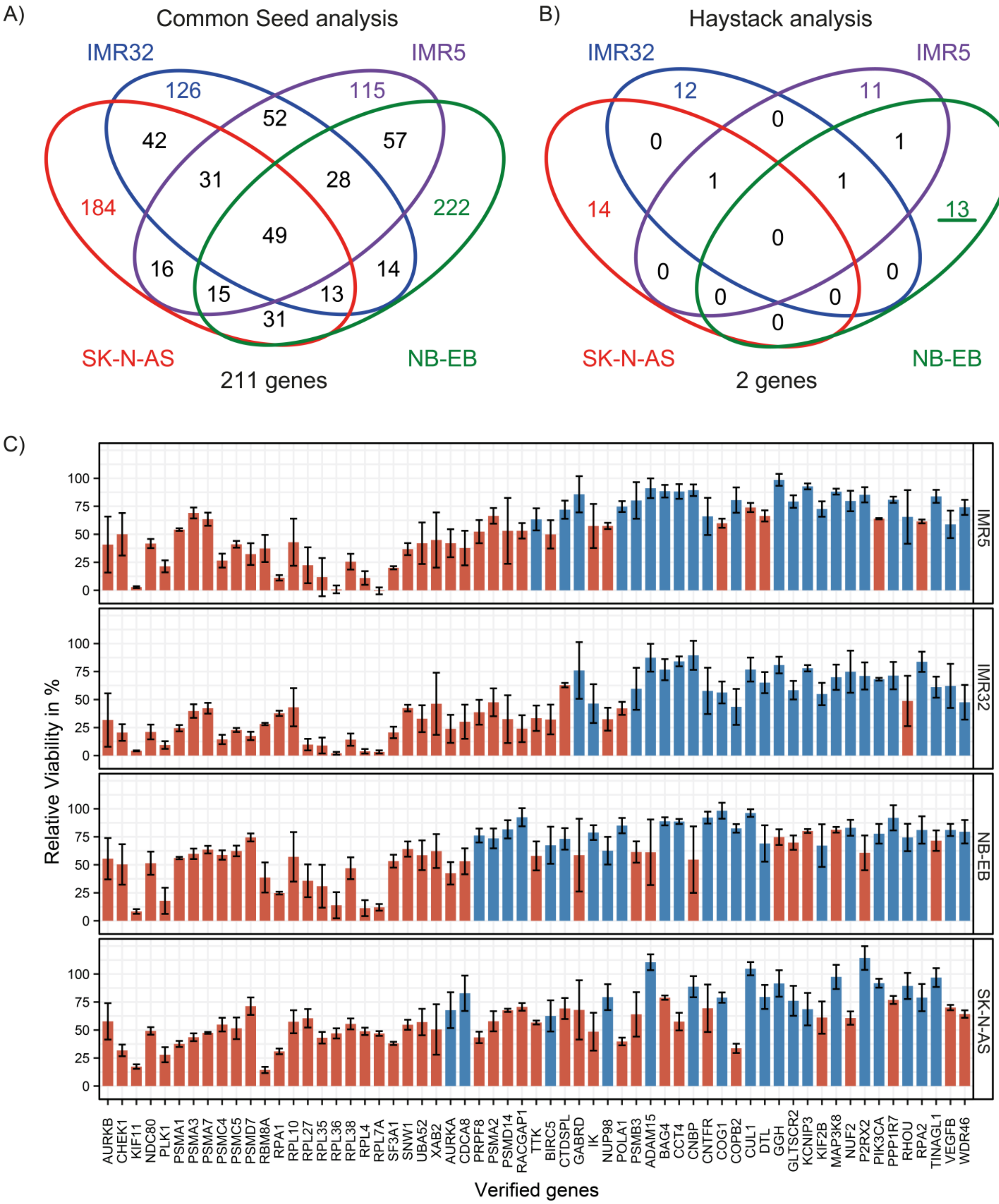

Supplementary figure S2: Venn diagrams of the overlap of candidate genes between the cell lines as identified by Common Seed **(A)** and Haystack Analysis **(B)** in the discovery screen. **C)** mean viabilities resulting from siRNA-mediated knockdown of 61

candidate genes that were verified in at least one cell line. Error bars represent the standard error of the three independent siRNAs per gene. Genes that were considered verified by RSA analysis in each cell line are colored maroon; non-verified genes blue.

A)

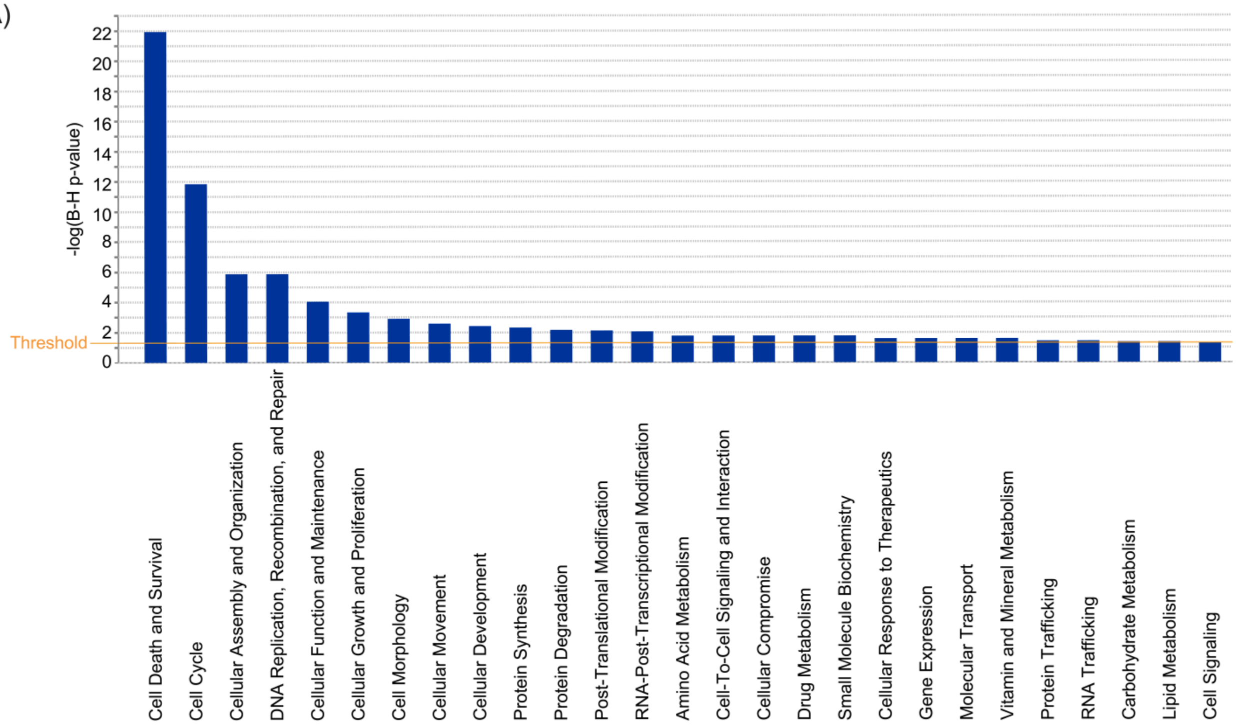

B)

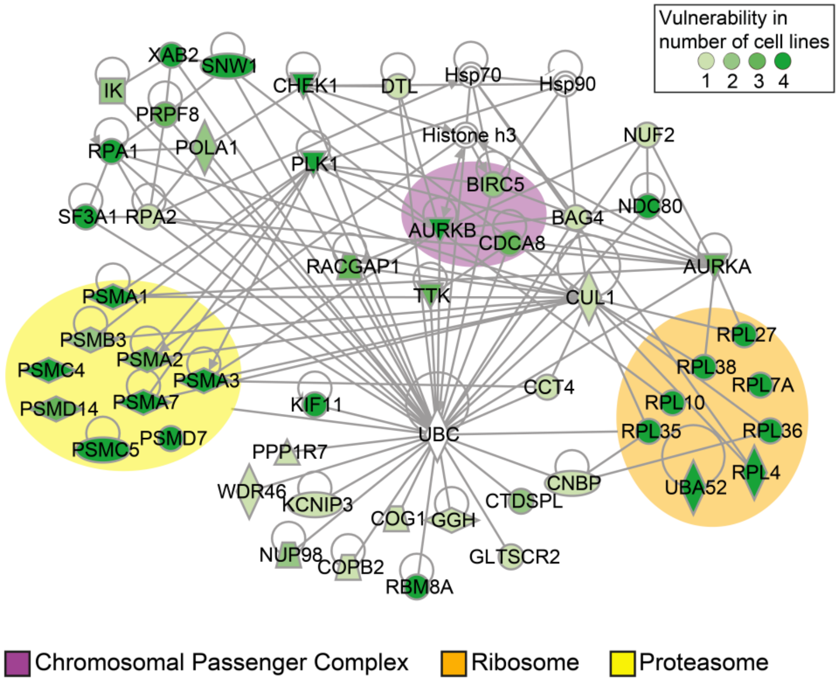

Supplementary figure S3: Vulnerability genes play a role in cell cycle progression and larger protein complexes. Ingenuity pathway analysis was conducted based on verification occurrence to annotate the 58 vulnerability genes in the secondary screen.

A) Molecular and cellular functions of the vulnerability genes. Blue bars represent the Benjamini-Hochberg-corrected significance scores for each function. The orange line indicates the negative $\log_{10}$ of the significance cutoff at -1.301. Molecular functions that did not pass the threshold are not displayed. **B)** Direct relationships between the genes of the two largest IPA networks. Symbols for the interaction partners are colored on a green scale based on the number of cell lines a gene appeared as candidate in. Genes and complexes without verification have a white background. Lines marking direct relationships between the subunits of the proteasome and the ribosome, respectively, were excluded for a better visualization.

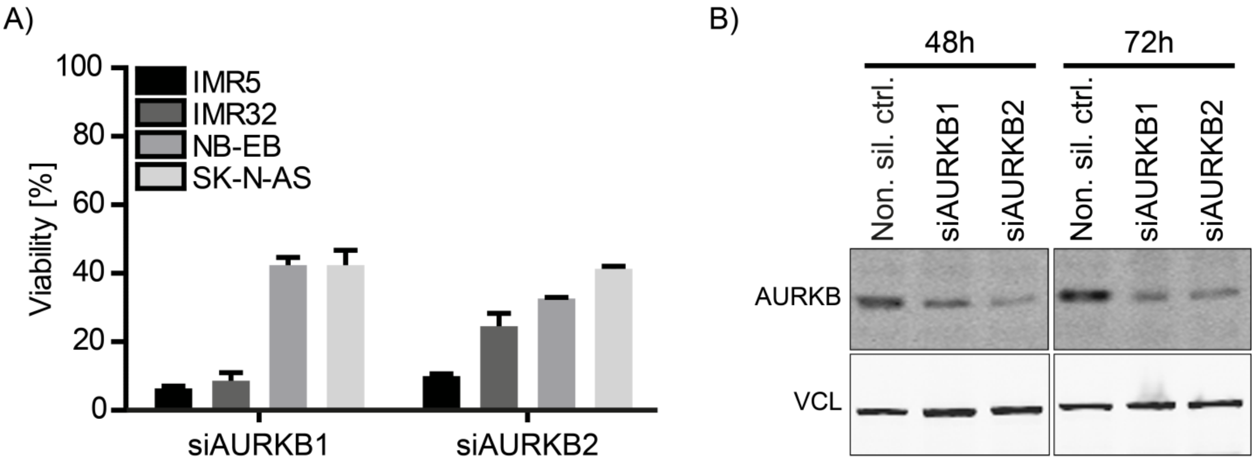

Supplementary figure S4: Verification of siRNA-mediated knockdown of AURKB.

A) Decrease of viability by the three siRNAs against AURKB in the four cell lines used in the verification screen. **B)** Western blots showing siRNA-mediated protein depletion of AURKB in IMR5. Cells were harvested at the indicated time points. Silencer Select Negative Control No. 2 siRNA served as the non-silencing control (Non. sil. ctrl.). Vinculin (VCL) was used as loading control.

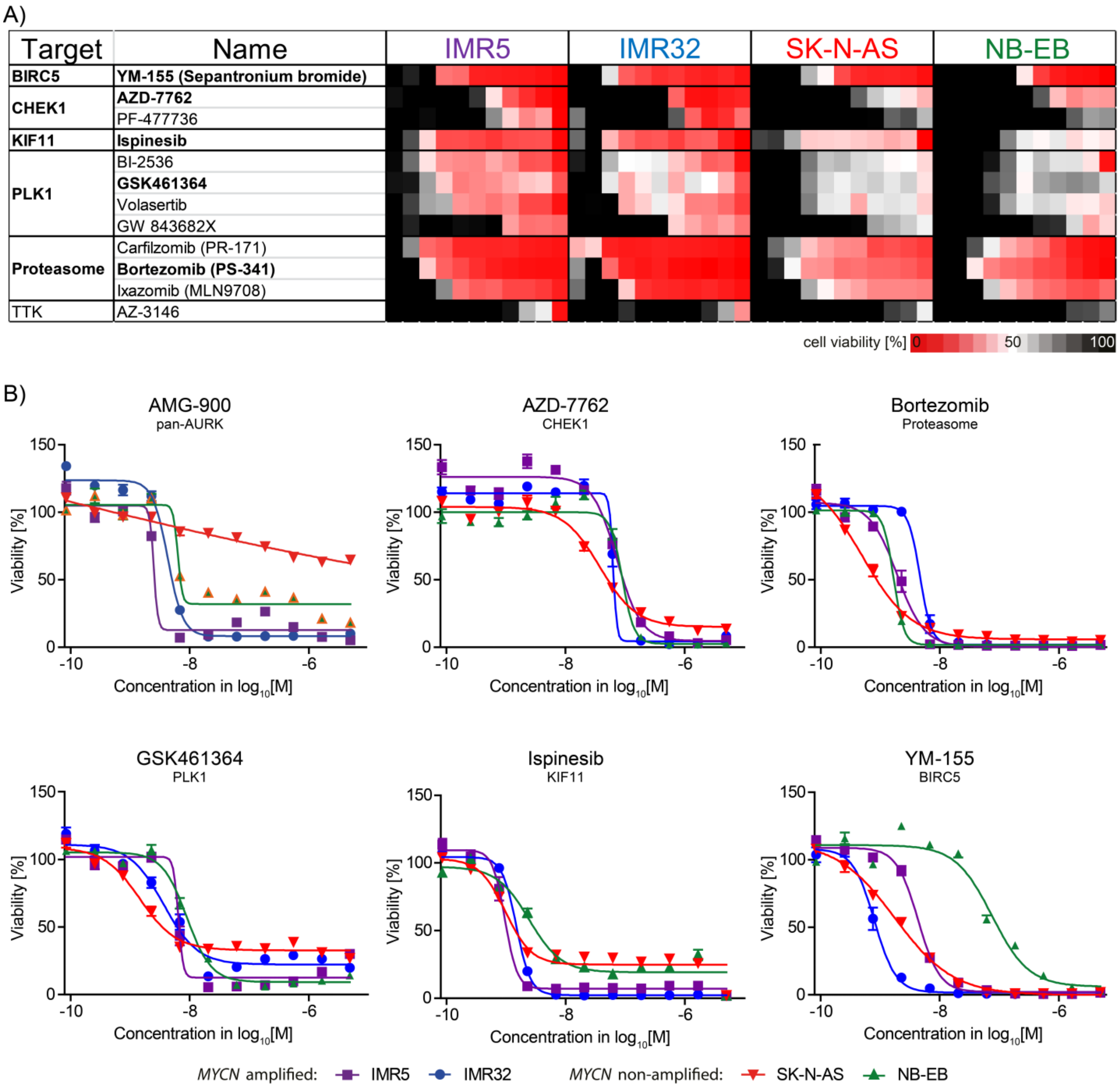

Supplementary figure S5: Drug response to the compounds contained in the MIPE 3.0 library targeting vulnerability genes identified by siRNA screening. A) heatmap of concentration-dependent effects on cell viability of targeting inhibitors. Drugs in bold were selected for retesting in 384-well plate assays. **B)** drug response curves of retested compounds in a 384-well format.

Supplementary table S1: siRNA screening data

Supplementary table S2: Drugs and classes in the MIPE library

NCGC SID	Name	MIPE3 Classification
NCGC00094717	6-mercaptopurine	Antimetabolite
NCGC00168784	Gemcitabine	Antimetabolite
NCGC00242485	Pemetrexed disodium	Antimetabolite
NCGC00263265	ASR-isobudimer-SO2Ph-4-CH2OC(O)NMe2	Antimicrobial
NCGC00263270	BTM-2C-dimer allyl oxime	Antimicrobial
NCGC00263268	BTM-2C-dimer ketone	Antimicrobial
NCGC00159337	Efavirenz	Antimicrobial
NCGC00159483	Hydroxychloroquine sulfate	Antimicrobial
NCGC00095055	Salinomycin	Antimicrobial
NCGC00092288	(-)-Blebbistatin	Antineoplastic
NCGC00025059	Actinomycin D	Antineoplastic
NCGC00263174	Ispinesib	Antineoplastic
NCGC00163411	Triptolide	Antineoplastic
NCGC00263271	Alisertib	Aurora Kinase
NCGC00263094	AMG-900	Aurora Kinase
NCGC00263089	AZD-1152-HQPA	Aurora Kinase
NCGC00263204	CYC-116	Aurora Kinase
NCGC00263203	Danusertib	Aurora Kinase
NCGC00263178	ENMD-981693	Aurora Kinase
NCGC00263151	JNJ-7706621	Aurora Kinase
NCGC00242482	SNS-314	Aurora Kinase
NCGC00168110	Tozasertib	Aurora Kinase
NCGC00242478	Alvespimycin hydrochloride	Chaperone
NCGC00161410	CCT-018159	Chaperone
NCGC00247877	CNF-2024	Chaperone
NCGC00183656	Elesclomol	Chaperone
NCGC00247878	NVP-AUY922	Chaperone
NCGC00263143	VER-155008	Chaperone
NCGC00263091	AT-7519	Cyclin-dependent Kinase
NCGC00263091	AT-7519	Cyclin-dependent Kinase
NCGC00242481	AZD-7762	Cyclin-dependent Kinase
NCGC00250401	Flavopiridol	Cyclin-dependent Kinase
NCGC00263129	PD-0332991	Cyclin-dependent Kinase
NCGC00263132	PF-477736	Cyclin-dependent Kinase

NCGC00263191	PHA-690509	Cyclin-dependent Kinase
NCGC00263168	PHA-793887	Cyclin-dependent Kinase
NCGC00025220	Purvalanol B	Cyclin-dependent Kinase
NCGC00094374	Seliciclib	Cyclin-dependent Kinase
NCGC00263167	SNS-032	Cyclin-dependent Kinase
NCGC00181170	Bendamustine	DNA Alkylating Agent
NCGC00015209	Cyclophosphamide	DNA Alkylating Agent
NCGC00090851	5-Azacitidine	DNA Epigenetic Machinery
NCGC00014891	RG-108	DNA Epigenetic Machinery
NCGC00168108	AG-14361	DNA Repair
NCGC00263096	Iniparib	DNA Repair
NCGC00238451	Olaparib	DNA Repair
NCGC00263173	Rucaparib	DNA Repair
NCGC00250404	Veliparib	DNA Repair
NCGC00242492	BIBR 1532	DNA Replication
NCGC00185090	NCGC00185090-04	DNA Replication
NCGC00189393	NCGC00189393-02	DNA Replication
NCGC00247866	NCGC00247866-02	DNA Replication
NCGC00263090	AZ-3146	Dual Specific Kinase
NCGC00250379	CT-99021	Dual Specific Kinase
NCGC00017363	cycloheximide	Dual Specific Kinase
NCGC00263183	MK-1775	Dual Specific Kinase
NCGC00189075	PD-0325901	Dual Specific Kinase
NCGC00188380	RDEA-119	Dual Specific Kinase
NCGC00263194	SB-216763	Dual Specific Kinase
NCGC00189073	Selumetinib	Dual Specific Kinase
NCGC00263187	TAK-733	Dual Specific Kinase
NCGC00263180	Trametinib	Dual Specific Kinase
NCGC00162405	ACPA	G-Coupled Protein Receptor
NCGC00263225	AG-041R	G-Coupled Protein Receptor
NCGC00181785	Aprepitant	G-Coupled Protein Receptor
NCGC00250393	CAP-232	G-Coupled Protein Receptor
NCGC00092384	Fenobam	G-Coupled Protein Receptor
NCGC00263107	GB 83	G-Coupled Protein Receptor
NCGC00025092	GR-159897	G-Coupled Protein Receptor
NCGC00167811	GW-9508	G-Coupled Protein Receptor
NCGC00263115	Ibutamoren mesilate	G-Coupled Protein Receptor
NCGC00250388	KRP 203	G-Coupled Protein Receptor
NCGC00092385	LY-320135	G-Coupled Protein Receptor
NCGC00183109	Maraviroc	G-Coupled Protein Receptor
NCGC00263122	MK-0354	G-Coupled Protein Receptor

NCGC00015682	MPEP	G-Coupled Protein Receptor
NCGC00263110	Osanetant	G-Coupled Protein Receptor
NCGC00263128	PD-0220245	G-Coupled Protein Receptor
NCGC00250378	Piboserod hydrochloride	G-Coupled Protein Receptor
NCGC00263139	Rolofylline	G-Coupled Protein Receptor
NCGC00015917	SB-206553	G-Coupled Protein Receptor
NCGC00015918	SB-224289	G-Coupled Protein Receptor
NCGC00242491	SB-265610	G-Coupled Protein Receptor
NCGC00025225	SCH-79797	G-Coupled Protein Receptor
NCGC00095150	Telmisartan	G-Coupled Protein Receptor
NCGC00025325	Vapiprost hydrochloride	G-Coupled Protein Receptor
NCGC00263108	Vorapaxar	G-Coupled Protein Receptor
NCGC00242479	Zibotentan	G-Coupled Protein Receptor
NCGC00242501	ITX3	GTPase
NCGC00263209	ML 141	GTPase
NCGC00092318	NSC 23766	GTPase
NCGC00263207	QS11	GTPase
NCGC00188866	SCHEMBL1461351	GTPase
NCGC00168109	Secin H3	GTPase
NCGC00250406	Tipifarnib	GTPase
NCGC00163474	Cyclopamine	Hedgehog Signaling
NCGC00263084	Hh-Ag1.5	Hedgehog Signaling
NCGC00263170	LY-2940680	Hedgehog Signaling
NCGC00250382	NVP-LDE-225	Hedgehog Signaling
NCGC00263210	SANT-2	Hedgehog Signaling
NCGC00242497	Vismodegib	Hedgehog Signaling
NCGC00263153	AR-42	Histone Epigenetic Machinery
NCGC00263155	Belinostat	Histone Epigenetic Machinery
NCGC00185850	BIX-01294	Histone Epigenetic Machinery
NCGC00165833	Entinostat	Histone Epigenetic Machinery
NCGC00242458	EX-527	Histone Epigenetic Machinery
NCGC00250390	ISOX	Histone Epigenetic Machinery
NCGC00250412	JQ1	Histone Epigenetic Machinery
NCGC00263121	MG-149	Histone Epigenetic Machinery
NCGC00263182	Mocetinostat	Histone Epigenetic Machinery
NCGC00183808	NCGC00183808-01	Histone Epigenetic Machinery
NCGC00241036	NCGC00241036-01	Histone Epigenetic Machinery
NCGC00263117	Panobinostat	Histone Epigenetic Machinery
NCGC00263136	Pracinostat	Histone Epigenetic Machinery
NCGC00263220	Romidepsin	Histone Epigenetic Machinery
NCGC00182052	SRT1720	Histone Epigenetic Machinery

NCGC00168085	Vorinostat	Histone Epigenetic Machinery
NCGC00186046	Caroverine hydrochloride	Ion Channel
NCGC00263016	DE-096	Ion Channel
NCGC00092329	Eliprodil	Ion Channel
NCGC00263106	Gavestinel sodium	Ion Channel
NCGC00250409	GSK-1016790A	Ion Channel
NCGC00263113	GYKI-53655	Ion Channel
NCGC00263214	HC-067047	Ion Channel
NCGC00024929	ICI-D7288	Ion Channel
NCGC00181343	Ivabradine hydrochloride	Ion Channel
NCGC00242480	Ivacaftor	Ion Channel
NCGC00163135	Niguldipine	Ion Channel
NCGC00015882	Riluzole	Ion Channel
NCGC00025379	SR 33805	Ion Channel
NCGC00016083	Verapamil	Ion Channel
NCGC00018204	Vinpocetine	Ion Channel
NCGC00159544	Zaldaride maleate	Ion Channel
NCGC00263083	Dalcetrapib	Lipid Metabolism
NCGC00263224	D-NMAPPD	Lipid Metabolism
NCGC00095134	Ezetimibe	Lipid Metabolism
NCGC00263228	FASN BI	Lipid Metabolism
NCGC00263230	FASN MRK	Lipid Metabolism
NCGC00164604	Fluvastatin	Lipid Metabolism
NCGC00263281	GSK-1995010	Lipid Metabolism
NCGC00263229	GSK837149A	Lipid Metabolism
NCGC00250374	Licofelone	Lipid Metabolism
NCGC00162249	MK-886	Lipid Metabolism
NCGC00037850	NCGC00037850	Lipid Metabolism
NCGC00185053	NCGC00185053	Lipid Metabolism
NCGC00263217	NVP231	Lipid Metabolism
NCGC00263131	PF-3845	Lipid Metabolism
NCGC00017324	Simvastatin	Lipid Metabolism
NCGC00025323	Ubenimex	Lipid Metabolism
NCGC00250411	Veliflapon	Lipid Metabolism
NCGC00159453	Zileuton	Lipid Metabolism
NCGC00263017	CPG-52364	Lipopolysaccharide Signaling
NCGC00263015	NCGC00263015-01	Lipopolysaccharide Signaling
NCGC00092381	ABT-702	Metabolite Conversion
NCGC00263235	ACC1 BMS	Metabolite Conversion
NCGC00263222	CAY10581	Metabolite Conversion
NCGC00022567	Cladribine	Metabolite Conversion

NCGC00263130	cPEPCK inhibitor	Metabolite Conversion
NCGC00182868	Daporinad	Metabolite Conversion
NCGC00024631	EBPC	Metabolite Conversion
NCGC00263221	Ezatiostat	Metabolite Conversion
NCGC00263232	GLS968	Metabolite Conversion
NCGC00165782	GW 4869	Metabolite Conversion
NCGC00263234	IDH-001	Metabolite Conversion
NCGC00263233	IPFK2	Metabolite Conversion
NCGC00274068	Itraconazole	Metabolite Conversion
NCGC00263211	JK 184	Metabolite Conversion
NCGC00263237	JZL-184	Metabolite Conversion
NCGC00015609	Lonidamine	Metabolite Conversion
NCGC00162423	Mithramycin	Metabolite Conversion
NCGC00185916	NCGC00185916	Metabolite Conversion
NCGC00186528	NCGC00186528	Metabolite Conversion
NCGC00238624	NCGC00238624	Metabolite Conversion
NCGC00262689	NCGC00262689	Metabolite Conversion
NCGC00182045	Pentostatin	Metabolite Conversion
NCGC00263135	Piraxostat	Metabolite Conversion
NCGC00182058	Doxercalciferol	Metabolites/Vitamins
NCGC00242511	Seocalcitol	Metabolites/Vitamins
NCGC00250387	KU 0060648	Nuclear Kinase
NCGC00242487	NCGC00242487	Nuclear Kinase
NCGC00250377	NU-7441	Nuclear Kinase
NCGC00250385	XMD8-92	Nuclear Kinase
NCGC00242477	AC-261066	Nuclear Receptor Transcription Factor
NCGC00092284	AHPN	Nuclear Receptor Transcription Factor
NCGC00263150	Andarine	Nuclear Receptor Transcription Factor
NCGC00182055	Bazedoxifene	Nuclear Receptor Transcription Factor
NCGC00167977	Bicalutamide	Nuclear Receptor Transcription Factor
NCGC00163107	CITCO	Nuclear Receptor Transcription Factor
NCGC00242504	Cortivazol	Nuclear Receptor Transcription Factor
NCGC00242505	Deacetyl cortivazol	Nuclear Receptor Transcription Factor
NCGC00091019	Dexamethasone	Nuclear Receptor Transcription

		Factor
NCGC00090752	Fenretinide	Nuclear Receptor Transcription Factor
NCGC00263111	GSK-3787	Nuclear Receptor Transcription Factor
NCGC00242476	GSK-4112	Nuclear Receptor Transcription Factor
NCGC00092344	GW-0742	Nuclear Receptor Transcription Factor
NCGC00167739	GW-4064X	Nuclear Receptor Transcription Factor
NCGC00241455	GW-501516	Nuclear Receptor Transcription Factor
NCGC00015480	GW-7647	Nuclear Receptor Transcription Factor
NCGC00022848	Hydrocortisone	Nuclear Receptor Transcription Factor
NCGC00242489	LG100268	Nuclear Receptor Transcription Factor
NCGC00263120	MDV-3100	Nuclear Receptor Transcription Factor
NCGC00025179	Mifepristone	Nuclear Receptor Transcription Factor
NCGC00263123	MK-767	Nuclear Receptor Transcription Factor
NCGC00163128	Pioglitazone HCl	Nuclear Receptor Transcription Factor
NCGC00015205	PK-11195	Nuclear Receptor Transcription Factor
NCGC00263137	RD-162	Nuclear Receptor Transcription Factor
NCGC00081778	SID 7969543	Nuclear Receptor Transcription Factor
NCGC00024928	Tamoxifen	Nuclear Receptor Transcription Factor
NCGC00161599	Troglitazone	Nuclear Receptor Transcription Factor
NCGC00263105	Turofexorate isopropyl	Nuclear Receptor Transcription Factor
NCGC00263093	Apilimod	Other
NCGC00092289	DA-3003-1	Other
NCGC00161825	GSK-3965	Other
NCGC00242512	methyl jasmonate	Other

NCGC00016925	Omeprazole	Other
NCGC00015870	Quercetine	Other
NCGC00024901	BD-1047	Other Receptor
NCGC00263126	Cutamesine hydrochloride	Other Receptor
NCGC00165875	PB-28	Other Receptor
NCGC00161408	anagrelide	Phosphodiesterase
NCGC00263226	BAY-60-7550	Phosphodiesterase
NCGC00250389	CDIBA	Phosphodiesterase
NCGC00263118	Lirimilast	Phosphodiesterase
NCGC00168459	NCGC00168459-01	Phosphodiesterase
NCGC00249759	NCGC00249759-01	Phosphodiesterase
NCGC00250085	NVP-ABE171	Phosphodiesterase
NCGC00162385	Olprinone	Phosphodiesterase
NCGC00095099	Sildenafil citrate	Phosphodiesterase
NCGC00025091	U-73122	Phosphodiesterase
NCGC00182037	Alacepril	Physiological Homeostasis
NCGC00091455	Celecoxib	Physiological Homeostasis
NCGC00160396	Pravadoline	Physiological Homeostasis
NCGC00345783	17β-hydroxy Wortmannin	PI3 Kinase
NCGC00263154	AZD-6482	PI3 Kinase
NCGC00263088	BAG-956	PI3 Kinase
NCGC00262604	BKM-120	PI3 Kinase
NCGC00262603	CAL-101	PI3 Kinase
NCGC00263223	CAY10626	PI3 Kinase
NCGC00025288	Deguelin	PI3 Kinase
NCGC00187482	GDC-0941	PI3 Kinase
NCGC00250408	GSK-2126458	PI3 Kinase
NCGC00168114	IC-87114	PI3 Kinase
NCGC00346503	Perifosine	PI3 Kinase
NCGC00187906	PI-103	PI3 Kinase
NCGC00238454	PIK-90	PI3 Kinase
NCGC00250392	AR-C155858	Plasma Membrane Transporter
NCGC00250402	Dapagliflozin	Plasma Membrane Transporter
NCGC00263219	Elacridar	Plasma Membrane Transporter
NCGC00263114	SCH-900435	Plasma Membrane Transporter
NCGC00247954	SSR-504734	Plasma Membrane Transporter
NCGC00263227	BAY-41-8543	Protease
NCGC00167803	GSI-9	Protease
NCGC00242503	Meclinertant	Protease
NCGC00263184	MK-0752	Protease
NCGC00262398	NCGC00262398	Protease

NCGC00263127	PD-166793	Protease
NCGC00263162	RO-4929097	Protease
NCGC00167751	SSR-69071	Protease
NCGC00263218	Teriflunomide	Protease
NCGC00263142	Tiplasinin	Protease
NCGC00263175	Tosedostat	Protease
NCGC00263188	YO-01027	Protease
NCGC00242506	Bortezomib	Proteasome
NCGC00249613	Carfilzomib	Proteasome
NCGC00249611	MLN-2238	Proteasome
NCGC00016423	(-)-Gossypol	Regulators of Apoptosis
NCGC00263280	Ac-SAH-p53-8	Regulators of Apoptosis
NCGC00015225	Chelerythrine chloride	Regulators of Apoptosis
NCGC00014873	HLI-373989	Regulators of Apoptosis
NCGC00167805	Ivachtin	Regulators of Apoptosis
NCGC00188344	Navitoclax	Regulators of Apoptosis
NCGC00092372	Necrostatin-1	Regulators of Apoptosis
NCGC00263124	Nutlin-3	Regulators of Apoptosis
NCGC00263166	Obatoclax	Regulators of Apoptosis
NCGC00167785	PAC-1	Regulators of Apoptosis
NCGC00242514	Sepantronium bromide	Regulators of Apoptosis
NCGC00263171	Serdemetan	Regulators of Apoptosis
NCGC00263212	SJ-172550	Regulators of Apoptosis
NCGC00263236	SM-164	Regulators of Apoptosis
NCGC00263208	TW-37	Regulators of Apoptosis
NCGC00165721	A 83-01	Serine/Threonine Kinase
NCGC00263147	A-674563	Serine/Threonine Kinase
NCGC00250373	A-769662	Serine/Threonine Kinase
NCGC00165736	Acadesine	Serine/Threonine Kinase
NCGC00263179	AR-00341677	Serine/Threonine Kinase
NCGC00250380	AZ-628	Serine/Threonine Kinase
NCGC00250391	AZD-7545	Serine/Threonine Kinase
NCGC00250405	AZD-8055	Serine/Threonine Kinase
NCGC00187481	BEZ-235	Serine/Threonine Kinase
NCGC00253438	BI-2536	Serine/Threonine Kinase
NCGC00263145	BI-78D3	Serine/Threonine Kinase
NCGC00263086	BI-D1870	Serine/Threonine Kinase
NCGC00263197	BMS-5	Serine/Threonine Kinase
NCGC00250386	BX-795	Serine/Threonine Kinase
NCGC00250386	BX-795	Serine/Threonine Kinase
NCGC00250407	CHIR-265	Serine/Threonine Kinase

NCGC00263099	CP-466722	Serine/Threonine Kinase
NCGC00242498	D-4476	Serine/Threonine Kinase
NCGC00241104	Doramapimod	Serine/Threonine Kinase
NCGC00165869	Dorsomorphin	Serine/Threonine Kinase
NCGC00238452	Enzastaurin	Serine/Threonine Kinase
NCGC00242507	Everolimus	Serine/Threonine Kinase
NCGC00263239	FMK	Serine/Threonine Kinase
NCGC00263109	GDC-0980	Serine/Threonine Kinase
NCGC00241982	GSK-269962A	Serine/Threonine Kinase
NCGC00263112	GSK-461364A	Serine/Threonine Kinase
NCGC00250410	GSK-650394	Serine/Threonine Kinase
NCGC00263181	GSK-690693	Serine/Threonine Kinase
NCGC00242217	GW 843682X	Serine/Threonine Kinase
NCGC00167767	IKK16	Serine/Threonine Kinase
NCGC00165811	IMD-0354	Serine/Threonine Kinase
NCGC00186035	IRAK-1-4 Inhibitor I	Serine/Threonine Kinase
NCGC00250383	Irestatin 9389	Serine/Threonine Kinase
NCGC00163381	KN-62	Serine/Threonine Kinase
NCGC00250376	KN-93	Serine/Threonine Kinase
NCGC00250396	KU-0063794	Serine/Threonine Kinase
NCGC00263190	KU-0064	Serine/Threonine Kinase
NCGC00229735	KU-60019	Serine/Threonine Kinase
NCGC00242598	LY2109761	Serine/Threonine Kinase
NCGC00249388	LY2157299	Serine/Threonine Kinase
NCGC00253463	MAP3K7 inhibitor	Serine/Threonine Kinase
NCGC00016564	Metformin HCl	Serine/Threonine Kinase
NCGC00241102	Midostaurin	Serine/Threonine Kinase
NCGC00186465	MK-2206	Serine/Threonine Kinase
NCGC00263021	MLN-120B	Serine/Threonine Kinase
NCGC00241410	NCGC00241410-01	Serine/Threonine Kinase
NCGC00241411	NCGC00241411-01	Serine/Threonine Kinase
NCGC00250395	OSI-027	Serine/Threonine Kinase
NCGC00263231	PDHK RIKEN	Serine/Threonine Kinase
NCGC00250398	PF-05212384	Serine/Threonine Kinase
NCGC00263213	PF-184	Serine/Threonine Kinase
NCGC00263134	PF-4708671	Serine/Threonine Kinase
NCGC00263165	PH-797804	Serine/Threonine Kinase
NCGC00250384	PHA-408	Serine/Threonine Kinase
NCGC00242499	Pim 1 inhibitor 2	Serine/Threonine Kinase
NCGC00187911	PLX-4720	Serine/Threonine Kinase
NCGC00165873	PS-1145	Serine/Threonine Kinase

NCGC00263119	Ruboxistaurin mesilate	Serine/Threonine Kinase
NCGC00241112	RWJ-67657	Serine/Threonine Kinase
NCGC00263196	S6K-18	Serine/Threonine Kinase
NCGC00015448	Salirasib	Serine/Threonine Kinase
NCGC00025035	SB-203580	Serine/Threonine Kinase
NCGC00025230	SB-431542	Serine/Threonine Kinase
NCGC00186024	SB-525334	Serine/Threonine Kinase
NCGC00165898	SD-208	Serine/Threonine Kinase
NCGC00263125	SG-00529	Serine/Threonine Kinase
NCGC00263186	SGI-1776	Serine/Threonine Kinase
NCGC00263192	Silmitasertib	Serine/Threonine Kinase
NCGC00242054	SJN 2511	Serine/Threonine Kinase
NCGC00263095	Sotrastaurin	Serine/Threonine Kinase
NCGC00263146	SR-3306	Serine/Threonine Kinase
NCGC00263141	STF-083010	Serine/Threonine Kinase
NCGC00263140	Talmapimod	Serine/Threonine Kinase
NCGC00263215	Torin-1	Serine/Threonine Kinase
NCGC00263216	Torin-2	Serine/Threonine Kinase
NCGC00250399	Vemurafenib	Serine/Threonine Kinase
NCGC00263087	Volasertib	Serine/Threonine Kinase
NCGC00249684	VX-702	Serine/Threonine Kinase
NCGC00241111	VX-745	Serine/Threonine Kinase
NCGC00242484	WYE-354	Serine/Threonine Kinase
NCGC00092276	Y-27632	Serine/Threonine Kinase
NCGC00025300	2-Methoxyestradiol	Signal Sensing Transcription Factor
NCGC00186460	Bardoxolone methyl	Signal Sensing Transcription Factor
NCGC00161703	NCGC00161703	Signal Sensing Transcription Factor
NCGC00263020	NCGC00263020-01	Signal Sensing Transcription Factor
NCGC00090903	Sulfasalazine	Signal Sensing Transcription Factor
NCGC00263019	WAY-204688	Signal Sensing Transcription Factor
NCGC00263238	Withaferin A	Signal Sensing Transcription Factor
NCGC00263148	Abiraterone	Steroid Biosynthesis
NCGC00015110	Aminoglutethimide	Steroid Biosynthesis
NCGC00164380	Formestane	Steroid Biosynthesis
NCGC00016973	Letrozole	Steroid Biosynthesis
NCGC00263092	Apratastat	TNF-alpha
NCGC00167491	Lenalidomide	TNF-alpha
NCGC00024415	Doxorubicin	Topoisomerase
NCGC00022001	Podofilox	Topoisomerase
NCGC00014925	Topotecan hydrochloride	Topoisomerase
NCGC00263098	Combretastatin A-4	Tubulin Polymerization

NCGC00181306	Docetaxel	Tubulin Polymerization
NCGC00263169	Plinabulin	Tubulin Polymerization
NCGC00163700	Vincristine sulfate	Tubulin Polymerization
NCGC00263149	AEE-788	Tyrosine Kinase
NCGC00185000	Afatinib	Tyrosine Kinase
NCGC00263206	AMG-47a	Tyrosine Kinase
NCGC00263201	AMG-51	Tyrosine Kinase
NCGC00263199	AMG-Tie2-1	Tyrosine Kinase
NCGC00263158	Amuvatinib	Tyrosine Kinase
NCGC00263195	AV-412	Tyrosine Kinase
NCGC00241108	Axitinib	Tyrosine Kinase
NCGC00250381	AZ-23	Tyrosine Kinase
NCGC00242486	AZD-1480	Tyrosine Kinase
NCGC00229512	BMS-509744	Tyrosine Kinase
NCGC00263157	BMS-777607	Tyrosine Kinase
NCGC00263176	BMS-794833	Tyrosine Kinase
NCGC00241107	Bosutinib	Tyrosine Kinase
NCGC00263160	Brivanib	Tyrosine Kinase
NCGC00263164	Cabozantinib	Tyrosine Kinase
NCGC00182713	Canertinib	Tyrosine Kinase
NCGC00263097	Cediranib	Tyrosine Kinase
NCGC00263101	CP-724714	Tyrosine Kinase
NCGC00250400	Crizotinib	Tyrosine Kinase
NCGC00263177	CUDC-101	Tyrosine Kinase
NCGC00244257	CYT387	Tyrosine Kinase
NCGC00263185	Dacomitinib	Tyrosine Kinase
NCGC00181129	Dasatinib	Tyrosine Kinase
NCGC00263172	DCC-2036	Tyrosine Kinase
NCGC00263102	Degrasyn	Tyrosine Kinase
NCGC00249685	Dovitinib	Tyrosine Kinase
NCGC00164574	Erlotinib hydrochloride	Tyrosine Kinase
NCGC00263104	Foretinib	Tyrosine Kinase
NCGC00159455	Gefitinib	Tyrosine Kinase
NCGC00253439	GSK-1904529A	Tyrosine Kinase
NCGC00159456	Imatinib	Tyrosine Kinase
NCGC00263189	JNJ-38877605	Tyrosine Kinase
NCGC00263193	KI-20227	Tyrosine Kinase
NCGC00167507	Lapatinib	Tyrosine Kinase
NCGC00249389	LDN-193189	Tyrosine Kinase
NCGC00015610	Leflunomide	Tyrosine Kinase
NCGC00263198	Lenvatinib	Tyrosine Kinase

NCGC00244256	Lestaurtinib	Tyrosine Kinase
NCGC00250403	Linifanib	Tyrosine Kinase
NCGC00250375	Linsitinib	Tyrosine Kinase
NCGC00241103	Masitinib	Tyrosine Kinase
NCGC00263205	Motesanib	Tyrosine Kinase
NCGC00188382	NCGC00188382-01	Tyrosine Kinase
NCGC00244250	NCGC00244250-01	Tyrosine Kinase
NCGC00263018	NCGC00263018-01	Tyrosine Kinase
NCGC00241101	Neratinib	Tyrosine Kinase
NCGC00183285	Nilotinib	Tyrosine Kinase
NCGC00263202	NVP-BSK805	Tyrosine Kinase
NCGC00263100	OSI-632	Tyrosine Kinase
NCGC00263159	OSI-930	Tyrosine Kinase
NCGC00188865	Pazopanib	Tyrosine Kinase
NCGC00187912	PCI-32765	Tyrosine Kinase
NCGC00242490	PD-166285	Tyrosine Kinase
NCGC00165863	PD-173074	Tyrosine Kinase
NCGC00263103	Pelitinib	Tyrosine Kinase
NCGC00263161	PF-04217903	Tyrosine Kinase
NCGC00242495	PF-431396	Tyrosine Kinase
NCGC00263133	PF-573228	Tyrosine Kinase
NCGC00346642	PHA-665752	Tyrosine Kinase
NCGC00263152	Ponatinib	Tyrosine Kinase
NCGC00253909	PRT-060318	Tyrosine Kinase
NCGC00242493	Quizartinib	Tyrosine Kinase
NCGC00182051	R406	Tyrosine Kinase
NCGC00263138	Regorafenib	Tyrosine Kinase
NCGC00263200	RO495	Tyrosine Kinase
NCGC00244253	Ruxolitinib	Tyrosine Kinase
NCGC00241099	Saracatinib	Tyrosine Kinase
NCGC00249346	SB1518	Tyrosine Kinase
NCGC00263163	SGX-523	Tyrosine Kinase
NCGC00167488	Sorafenib	Tyrosine Kinase
NCGC00238453	TAE-684	Tyrosine Kinase
NCGC00241097	Tandutinib	Tyrosine Kinase
NCGC00249392	Telatinib	Tyrosine Kinase
NCGC00244252	TG-101348	Tyrosine Kinase
NCGC00249390	Tivozanib	Tyrosine Kinase
NCGC00229511	Tofacitinib	Tyrosine Kinase
NCGC00167513	Vandetanib	Tyrosine Kinase
NCGC00263156	Vargatef	Tyrosine Kinase

NCGC00181350	Vatalanib	Tyrosine Kinase
NCGC00263144	WZ-4002	Tyrosine Kinase
NCGC00263116	ICG-001	Wnt Signaling
NCGC00250397	NVP-XAV-939	Wnt Signaling
NCGC00263085	Wnt-C59	Wnt Signaling

Target	Inhibitor	IMR32 IC_{50} [nM]	IMR32 norm. AUC [%]	IMR5 IC_{50} [nM]	IMR5 norm. AUC [%]	NB-EB IC_{50} [nM]	NB-EB norm. AUC [%]	SK-N-AS IC_{50} [nM]	SK-N-AS norm. AUC [%]
**pan-AURK**	**AMG-900**	4.29	39.49	2.50	40.48	6.32	57.57	0.39	75.64*
**BIRC5**	**YM-155**	0.80	21.95	4.35	36.85	75.98	64.64	1.78	29.57*
**CHEK1**	**AZD-7762**	63.38	62.25	72.97	62.66	89.83	64.80	37.79	61.81
**KIF11**	**Ispinesib**	1.49	27.40	0.95	27.45	2.42	45.39	0.98	42.02
**PLK1**	**GSK461364**	3.68	47.24	~6.47	44.54	8.96	47.25	1.46	48.39
**Proteasome**	**Bortezomib**	4.63	37.22	1.93	28.20	1.68	27.79	0.50	24.26*

Supplementary table S3: IC_{50} concentrations in nM and normalized area under the curve (AUC) in % of compounds targeting vulnerability genes. Assays were conducted in a 384-well plate format. AUC of drug response curves were calculated with GraphPad Prism and normalized against the predicted upper asymptote. * estimation of upper asymptote was incorrect due to incomplete curve profile; viability measured at the lowest drug concentration was used as a surrogate.
